# TSH Ligand‐Based CAR‐T Cell Effectively Eradicates TSHR‐Positive Thyroid Cancer with Favorable Safety Profile

**DOI:** 10.1002/advs.202513243

**Published:** 2025-09-23

**Authors:** Fei Wang, Hui Zuo, Li Liu, Shiyuan Wang, Haifang Zhao, Zhao Liu, Gang Wang, Zhao Liu, Junnian Zheng, Chuan Xu, Hongwei Du

**Affiliations:** ^1^ Cancer Institute Xuzhou Medical University 209 Tongshan Road Xuzhou Jiangsu 221004 China; ^2^ Center of Clinical Oncology the Affiliated Hospital of Xuzhou Medical University 99 Huaihai Road Xuzhou Jiangsu 221002 China; ^3^ Jiangsu Center for the Collaboration and Innovation of Cancer Biotherapy Xuzhou Medical University 209 Tongshan Road Xuzhou Jiangsu 221004 China; ^4^ Department of General Surgery the Affiliated Hospital of Xuzhou Medical University 99 Huaihai Road Xuzhou 221002 China; ^5^ Department of Nuclear Medicine The Affiliated Hospital of Xuzhou Medical University 99 West Huaihai Road Xuzhou Jiangsu 221002 China; ^6^ Department of Oncology & Cancer Institute Sichuan Academy of Medical Sciences Sichuan Provincial People's Hospital University of Electronic Science and Technology of China Chengdu 610072 China; ^7^ Jinfeng Laboratory Chongqing 401329 China

**Keywords:** CAR‐T, cell therapy, differentiated thyroid cancer, TSHR, TSH

## Abstract

CAR‐T therapy faces significant challenges in solid tumors, including the shortage of targetable antigen and the immunogenicity of CAR molecules. Here, TSHR is identified as specifically expressed in DTC, but absent in other normal tissues, making it an ideal target for CAR‐T therapy. To overcome CAR immunogenicity, a novel CAR molecule is engineered using TSH (TSH‐CAR), the natural ligand of TSHR, as the antigen‐binding domain to target TSHR. The TSH‐CAR‐T cells demonstrate effective antitumor activity against TSHR‐positive differentiated thyroid cancer (DTC) cell lines in vitro, accompanied by cytokine release (IFNγ, IL‐2) and robust proliferation. In addition, TSH‐CAR‐T cells achieved complete tumor eradication and sustained remission in two distinct thyroid cancer xenograft models. Furthermore, for comprehensively evaluate the safety profile of TSH‐CAR‐T cell, a murine TSH‐CAR (mTSH‐CAR‐T) is engineered, revealing that mTSH‐CAR‐T cells effectively control mTSHR‐positive tumor growth without evident on‐target/off‐tumor effect in immunocompetent syngeneic mouse tumor models, except for the transient and reversible impairment of thyroid follicles, which is acceptable given prior thyroidectomy in DTC patients. The potent preclinical efficacy and favorable safety profile strongly support the clinical translation of TSH‐CAR‐T for patients suffering from metastatic and radioiodine‐resistant DTC.

## Graphical abstract

TSHR is highly expressed in differentiated thyroid cancer (DTC), making it an ideal target for adoptive immune cell therapy. A novel Ligand‐based TSH‐CAR‐T cell approach effectively eradicated TSHR‐positive thyroid tumors in vitro and accomplished full remission in tumor models with favorable safety profile, highlighting that TSH‐CAR‐T cells could represent a promising new therapeutic option for patients with advanced DTC.

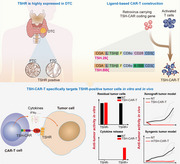

## 1 Introduction

Thyroid cancer is the most common endocrine system malignancy, and its occurrence is steadily increasing globally, having surged nearly threefold over the last three decades, the age of onset for thyroid cancer tends to be relatively younger.^[^ ^1^, ^2^, ^3^ ^]^ Thyroid cancer encompasses both differentiated and undifferentiated varieties, with DTC being the predominant form, representing over 90% of total cases.^[^ ^4^ ^]^ Although the 10‐year survival rate for DTC approaches 90% after surgical intervention followed by radioactive iodine therapy,^[^ ^5^ ^]^ between 7% and 23% of patients may experience distant metastasis, and among these, two‐thirds develop resistance to radioactive iodine therapy, resulting in a 10‐year survival rate of below 10%.^[^ ^6^, ^7^, ^8^, ^9^ ^]^ Given the high incidence of thyroid cancer, with more than 820 000 new cases reported globally each year, the annual mortality figures reach as high as 47 500, making it as one of the leading causes of cancer‐related death.^[^ ^10^ ^]^ Currently, there is an urgent need for new therapeutic strategy for the patients with recurrent, metastatic, and conventionally treatment‐resistant thyroid cancer.

Chimeric antigen receptor‐engineered T cell (CAR‐T) therapy has achieved great success in the treatment of blood cancers in clinical.^[^ ^11^, ^12^ ^]^ To date, 11 CAR‐T therapies were approved by FDA for treating various B‐cell malignancies and multiple myeloma.^[^ ^13^, ^14^ ^]^ The successful application of CAR‐T cell therapy in hematological cancers has promoted the considerable investigation on CAR‐T cell therapy for solid tumors. Nevertheless, the progress of CAR‐T therapy in solid tumor has been significantly slower compared to that in hematological malignancies.^[^ ^15^, ^16^ ^]^

A significant obstacle hindering the advancement of CAR‐T cell therapy for solid tumors is the absence of tumor specific antigens. Currently, most CAR‐T targets in solid tumors are tumor‐associated antigens, which are not exclusive to tumor tissue but are also found in normal tissues, such as MSLN, HER2, EGFR, and B7‐H3.^[^ ^17^, ^18^, ^19^, ^20^ ^]^ Thus, when these tumors are targeted, the normal tissues expressing the corresponding antigens are inevitably affected. This is why CAR‐T therapy may result in life‐threatening toxic effects known as “on‐target‐off‐tumor” repercussions.^[^ ^21^, ^22^, ^23^ ^]^

In this study, we identified thyroid‐stimulating hormone receptor (TSHR) as a promising antigen for CAR‐T cell therapy in thyroid cancer. This receptor, a glycoprotein, is widely present on the membrane of thyroid follicular epithelium,^[^ ^24^ ^]^ and play a crucial role in mediating the effects of thyroid‐stimulating hormone (TSH), which is secreted by the pituitary gland.^[^ ^25^ ^]^ It activates a G‐protein‐coupled signaling cascade, leading to the synthesis and release of thyroid hormones T3 and T4, which are essential for regulating metabolism and development.^[^ ^26^ ^]^

TSHR has been found to be highly expressed in DTC through IHC and qPCR,^[^ ^27^, ^28^, ^29^, ^30^ ^]^ more significantly, its expression was also detected in tumor tissues from metastatic and radioiodine‐resistant DTC patients.^[^ ^29^, ^31^, ^32^ ^]^ Additionally, considering that the primary clinical approach for thyroid cancer involves surgical removal of the tumor and the normal thyroid,^[^ ^33^, ^34^ ^]^ TSHR expression in normal thyroid tissue raises no concern for potential off‐tumor toxicity, as the normal thyroid has already been excised in patients. Taken together, this makes it a compelling target for CAR‐T therapy in thyroid cancer.

The immunogenicity associated with the scFv sequence contained within the CAR molecule could provoke the host's immune system to identify and eliminate CAR‐T cells, thereby hindering the long‐term persistence of CAR‐T cells, this represents another significant obstacle in the development of effective CAR‐T therapies for solid tumors.^[^ ^21^, ^35^ ^]^ Typically, the scFv derives‐ from murine monoclonal antibodies (mAbs). Although humanized antibodies can reduce immunogenicity, they are not members of house‐keeping proteins and cannot entirely eliminate immunogenicity.^[^ ^35^ ^]^

Here, we propose utilizing TSHR's natural ligand, TSH, as the antigen recognition domain for constructing a CAR against TSHR, which may completely bypass immunogenicity since it is a house‐keeping gene expressed native human protein. Therefore, TSH was tandemly linked with either the CD28 or 4‐1BB costimulatory domain to construct CAR molecule (TSH‐CAR), and we comprehensively investigated the anti‐tumor efficacy and safety of TSH‐CAR‐T cells both in in vitro and in vivo experiments. The results showed that TSH‐CAR‐T cells specifically target TSHR‐positive tumor cells and effectively control tumor growth in in vitro co‐culture experiments with thyroid cancer cells as well as in mouse tumor models. More importantly, using CAR‐T cells constructed with murine TSH (mTSH‐CAR‐T), we were able reliably and meticulously to investigate the potential safety profile of TSH‐CAR‐T cells within syngeneic mouse models, discovering that mTSH‐CAR‐T cells did not cause any “on‐target‐off‐tumor” adverse effects aside from thyroid tissue impairment. Our finding indicates the feasibility of employing TSH targeting TSHR via CAR‐T cells for treating advanced thyroid cancer, presenting a new therapeutic strategy for DTC patients facing recurrence, metastasis, and radioactive iodine therapy resistance.

## 2 Results

### 2.1 TSHR is Highly Expressed in Differentiated Thyroid Cancer but Absent in Normal Tissues

**Figure 1:**
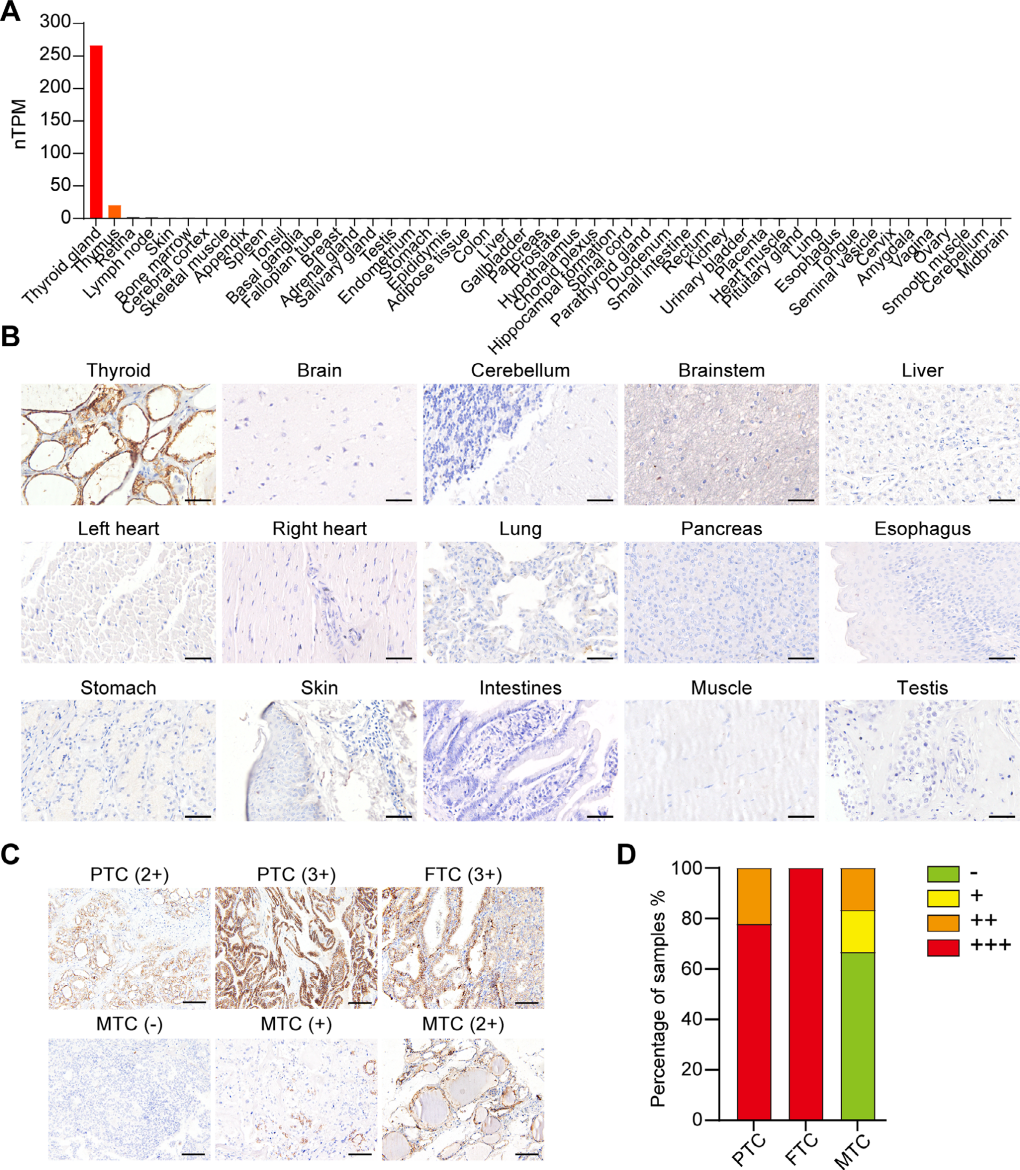
TSHR is highly expressed in differentiated thyroid cancer. A) Analyzing the mRNA expression level of TSHR in normal tissues from the human protein atlas database. B) The expression of TSHR protein in normal human tissues was examined via IHC. Scale bar: 100 µm. C) Representative TSHR expression levels in thyroid tumor sections demonstrating 3+ (strong), 2+ (moderate), + (weak), ‐ (none) via IHC staining. Scale bar: 100 µm. D) The percentage of thyroid tumor samples with different expression level of TSHR was quantified based on the intensity of TSHR in (C).

To examine if TSHR could serve as a potential target antigen for CAR‐T cell therapy in thyroid cancer, a detailed evaluation of the TSHR expression levels in normal human tissues and thyroid carcinoma samples was conducted. The RNA sequencing data from the Human Protein Altas indicated that TSHR mRNA is abundantly expressed in the normal thyroid gland, with undetectable expression in other normal tissues (**Figure** **1**A), apart from a minimal presence in plasma cells of thymus (Figure S1, Supporting Information). The immunohistochemical (IHC) staining results verified that TSHR is uniquely expressed in the thyroid gland and absent in other normal tissues (Figure 1B; Figure S2, Supporting Information), including the testis, which had been previously reported positive by other research groups.^[^ ^36^ ^]^

Furthermore, the IHC results revealed that TSHR is highly expressed in differentiated thyroid cancers, including both papillary (PTC) and follicular (FTC) thyroid carcinoma, while exhibiting comparatively low expression in medullary thyroid cancer (MTC) (Figure 1C,D), aligning with earlier reports from other researchers.^[^ ^32^ ^]^ These findings demonstrated that TSHR is a promising target for CAR‐T therapy in differentiated thyroid cancer.

### 2.2 Engineer CAR to Aim at TSHR via its Inherent Ligand TSH

**Figure 2:**
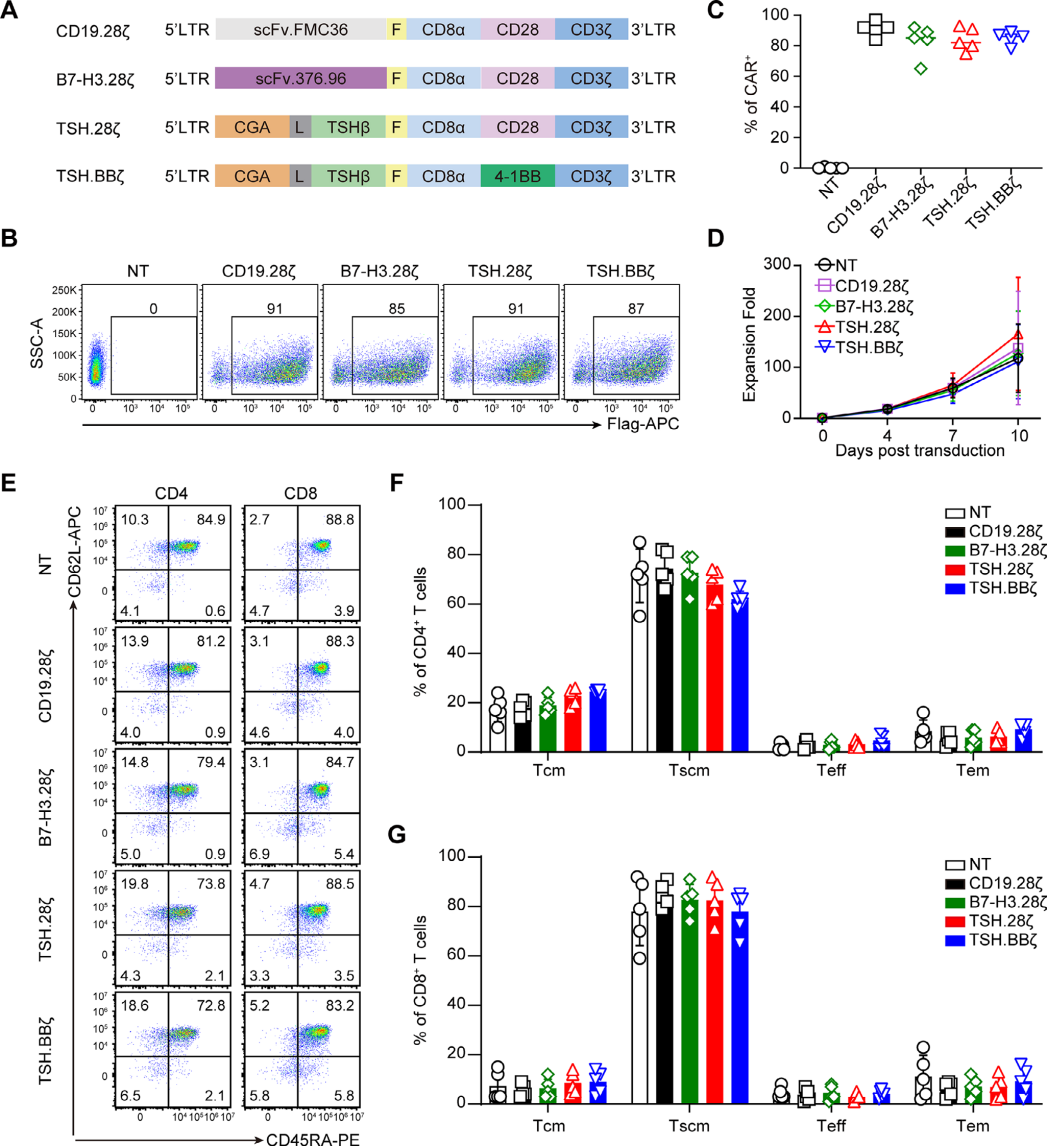
TSH‐CAR‐T cells exhibit comparable CAR expression level and memory phenotype with conventional CAR‐T cells. A) Schematic structure of the CAR constructs. scFv.376.96, scFv of the anti‐B7‐H3 mAb 376.96; scFv.FMC36, scFv of the anti‐CD19 mAb FMC36; CD8α, the hinge and transmembrane region of CD8α; CD28, intracellular domain of CD28; 4‐1BB, intracellular domain of 4‐1BB; CD3ζ, intracellular domain of CD3ζ; L, (G_4_S)_3_ linker; F, flag tag. B) Representative flow cytometry plots showing the transduction efficiency of CARs in their respective CAR‐T cells. C) Summary of the CARs transduction efficiency. Data were shown as individual values and the mean ± SD (*n* = 5). D) Expansion kinetics of the CAR‐T cells (*n* = 5). E) Representative flow cytometry plots showing expression of CD45RA and CD62L in the indicated CD4^+^ and CD8^+^ CAR‐T cells. F,G) Quantification of the frequency of stem cell memory T cells (Tscm, CD45RA^+^CD62L^+^), central memory T cells (Tcm, CD45RA^‐^CD62L^+^), effector memory T cells (Tem, CD45RA^‐^CD62L^‐^), and effector T cells (Teff, CD45RA^+^CD62L^‐^) in CD4^+^ (F) and CD8^+^ CAR‐T cells (G) (*n* = 5).

CAR molecules derived from mouse monoclonal antibodies may provoke immune response from the host's immune system, leading to the clearance of CAR‐T cells.^[^ ^34^ ^]^ To address this problem, we propose utilizing the TSHR's natural ligand‐TSH, as the antigen‐binding domain to target TSHR and fully avoid the immunogenicity. TSH, comprising two subunits, the universal subunit CGA and the specific subunit TSHβ, where CGA is shared with other glycoprotein hormones (e.g., HCG, LH, FSH), while TSHβ is exclusive to TSH and determines its biological specificity.^[^ ^37^ ^]^ Based on the precise binding of ligands to receptors, we constructed CGA‐TSHβ with a (G_4_S)_3_ linker (TSH) or reversely TSHβ‐CGA (TSH‐reverse) (Figure S3A, Supporting Information), as the antigen binding domain aimed at targeting TSHR, and followed by incorporating either CD28 or 4‐1BB costimulatory domain, and CD3ζ chain (**Figure** **2**A; Figure S3A, Supporting Information). In addition, the B7‐H3‐CAR and CD19‐CAR were used as positive and negative controls, respectively (Figure 2A). The transduction efficiency of the CARs of TSH‐28ζ, TSH‐BBζ, CD19‐CAR, and B7‐H3‐CAR exceeded 90%, with no significant difference between observed among them (Figure 2B,C; Figure S3B,C, Supporting Information). In addition, the expansion of TSH‐28ζ or TSH‐BBζ CAR‐T cells was comparable to that of control CAR‐T cells (Figure 2D), and memory phenotype analysis revealed comparable proportions of central‐memory (Tcm), effector‐memory cell (Tem) and stem cell memory in CD4 and CD8 cell populations across these CAR‐T cells (Figure 2E–G).

### 2.3 TSH‐CAR‐T Cells Show Effective Anti‐Tumor Activity Against TSHR‐Positive Tumor Cells

**Figure 3:**
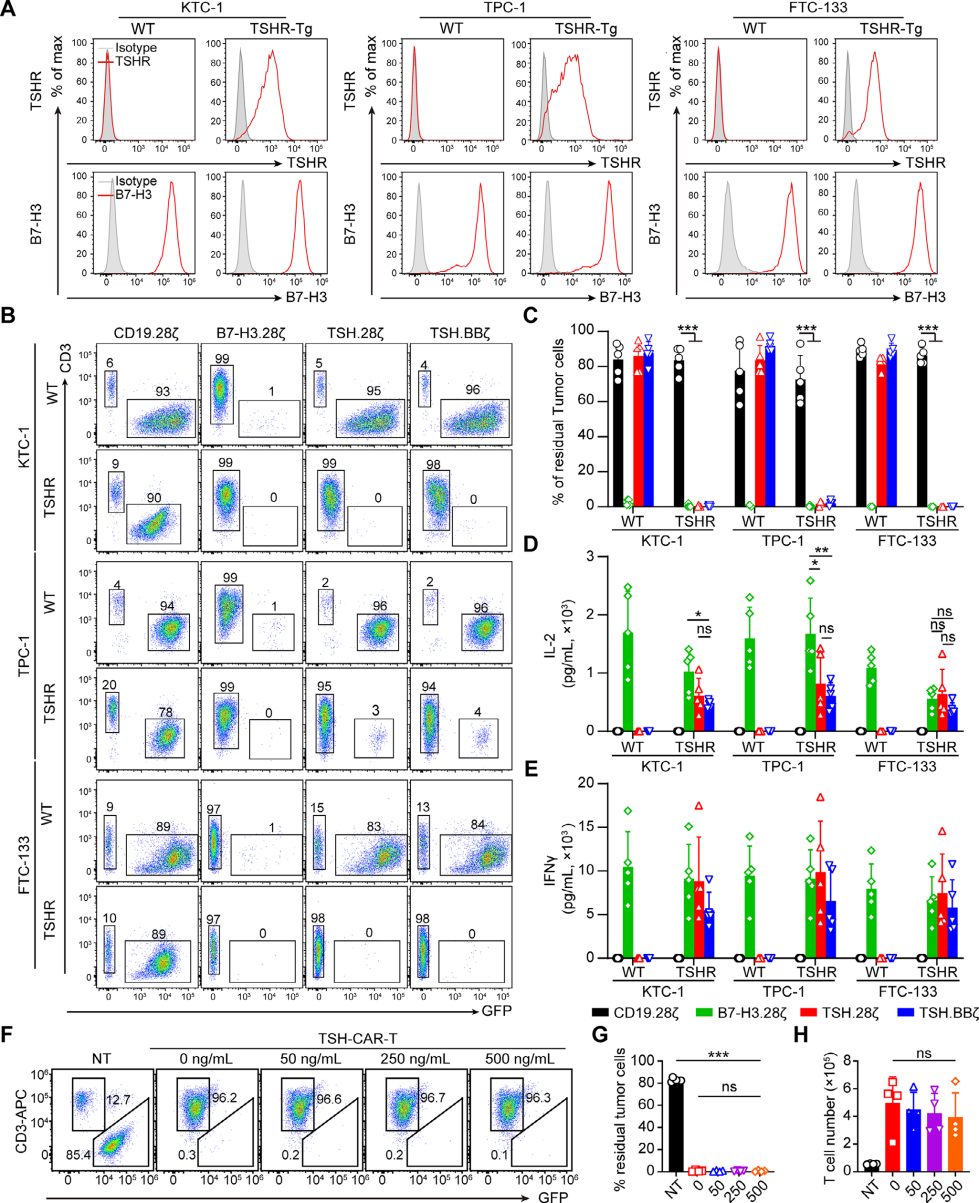
TSH‐CAR‐T specifically eliminate TSHR‐positive thyroid cancer cells. A) The representative expression pattern of TSHR and B7‐H3 in the DTC cell lines. TSHR‐Tg, TSHR‐transgenic. B,C) Tumor cells labeled with GFP were co‐cultured with CAR‐Ts at the T cell to tumor cell ratio of 1–5. On day 5, cancer cells (GFP^+^) and CAR‐T cells (CD3^+^) were enumerated by flow cytometry. Representative flow‐cytometry plots (B) and quantification of residual tumor cells (C) are illustrated (*n* = 5). Error bars denote SD. ***p < 0.001; one‐way ANOVA with Tukey's multiple comparisons test correction. D,E) Summary of IL‐2 (D) and IFNγ (E) released by the CAR‐T cells in the culture supernatant following 24 hours of co‐culturing with tumor cells (*n* = 5). Error bars denote SD. *p < 0.05, **p < 0.01; ns, not significant; one‐way ANOVA with Tukey's multiple comparisons test correction. F–H) KTC‐1‐TSHR cancer cells labeled with GFP were co‐cultured with NT or TSH‐CAR‐Ts in the presence of different concentration of TSH‐Fc protein, and the ratio of T cell to tumor cells was 1–5. On day 5, cancer cells (GFP^+^) and CAR‐T cells (CD3^+^) were enumerated by flow cytometry. Representative flow‐cytometry plots (F) and quantification of residual tumor cells (G) and T cells (H) are illustrated (*n* = 4). ***p < 0.001; ns, not significant; one‐way ANOVA with Tukey's multiple comparisons test with correction.

To identify TSHR‐positive DTC cell lines to serve as target cells for evaluating the specificity and anti‐tumor effectiveness of CAR‐T cells directed against TSHR, we examined the TSHR expression in the DTC cell lines, including TPC‐1, KTC‐1, and FTC‐133. However, none of these cell lines exhibited TSHR expression (**Figure** **3**A), which aligns with findings from other studies indicating that DTC tumor cells lack TSHR expression in vitro.^[^ ^32^, ^38^ ^]^ This absence may be attributed to the observation that the DTC cells tend to lose TSHR expression once they exit the tumor microenvironment,^[^ ^39^ ^]^ such as when cultured in vitro. Thus, we expressed TSHR exogenously into these three cancer cell lines to create TSHR‐positive target cells (Figure 3A). Additionally, we confirmed the presence of another pan‐solid tumor antigen, B7‐H3, in these cell lines (Figure 3A), thereby reinforcing the utilization of B7‐H3‐CAR‐T cells as a positive control.

The anti‐tumor activity of TSH‐CAR‐T cells was assessed by in vitro coculture experiment. Three DTC cell lines, including both the wild type and TSHR‐overexpressing variants of each cell line, were cocultured with CD19‐CAR‐T (negative control), B7‐H3‐CAR‐T (positive control), and TSH‐CAR‐T (TSH‐28ζ‐CAR‐T and TSH‐BBζ‐CAR‐T) and TSH‐reverse‐CAR‐T (TSH‐reverse‐28ζ‐CAR‐T and TSH‐reverse‐BBζ‐CAR‐T) cells at a ratio of 1:5 ratio for 5 days. The results showed that both TSH‐28ζ‐CAR‐T and TSH‐BBζ‐CAR‐T cells effectively eradicated the TSHR‐positive tumor cells compared to the CD19‐CAR‐T group, and the anti‐tumor capacity is comparable to B7‐H3‐CAR‐T cells (Figure 3B,C). In addition, TSH‐28ζ‐CAR‐T and TSH‐BBζ‐CAR‐T cells had no killing effect on WT tumor cells (Figure 3B,C). In addition, the TSH‐28ζ‐CAR‐T and TSH‐BBζ‐CAR‐T cells produced substantial amounts of IFNγ and IL‐2 when coculturing with TSHR‐positive tumor cells, while no cytokines were released in the coculture with WT tumor cells (Figure 3D,E). However, the TSH‐reverse‐CAR‐T cells didn't exhibit effective antitumor activity against TSHR‐positive tumor cells compare to TSH‐CAR‐T cells (Figure S3C,D, Supporting Information). Therefore, only the TSH‐CAR‐T cells were selected for further investigation.

Additionally, we examined the proliferation of TSH‐CAR‐T cells after activation by TSHR‐positive target cells using the carboxyfluorescein diacetate succinimidyl ester (CFSE) dilution assay. The results indicated that TSH‐CAR‐T cells exhibited a marked decrease in CFSE intensity post‐co‐culturing with tumor cells, signifying a robust proliferative response upon antigen engagement (Figure S4, Supporting Information).

To further evaluate whether free TSH in vivo might hinder the anti‐tumor efficacy of TSH‐CAR‐T cells, we added different concentration of TSH‐Fc protein into the co‐culture system and assessed its killing efficacy. The results showed that even at a concentration of 500 ng mL^−1^ TSH‐Fc, which far exceeds the physiological range (0.4–4.5 mIU L^−1^, ≈0.02–0.2 ng mL^−1^) of TSH found in human blood,^[^ ^40^ ^]^ TSH‐CAR‐T cells remain proficient in obliterating tumor cells (Figure 3F–H).

Since Zeng et al. reported that TSHR expression on T cells mediates T cell exhaustion,^[^ ^41^ ^]^ thus, it is necessary to validate the expression of TSHR on T cells and whether TSH‐CAR leads to T cell self‐recognition and mutual killing. We first examined the expression of TSHR on both activated and non‐activated T cells, and no TSHR expression was detected (Figure S5A, Supporting Information). Additionally, to further illustrate whether TSH‐CAR‐T cells have self‐killing effect, we co‐cultured activated and non‐activated GFP‐labeled T cells with NT and TSH‐CAR‐T cells respectively, measuring the proportion of GFP^+^ cells after 24 h. The results showed that TSH‐CAR‐T cells had no any killing effect on GFP^+^ cells. This finding indicated that TSH‐CAR‐T did not exhibit self‐killing effect (Figure S5B–D, Supporting Information).

### 2.4 TSH‐CAR‐T Cells Effectively Eradicate Tumor in Thyroid Cancer Xenograft Models

**Figure 4:**
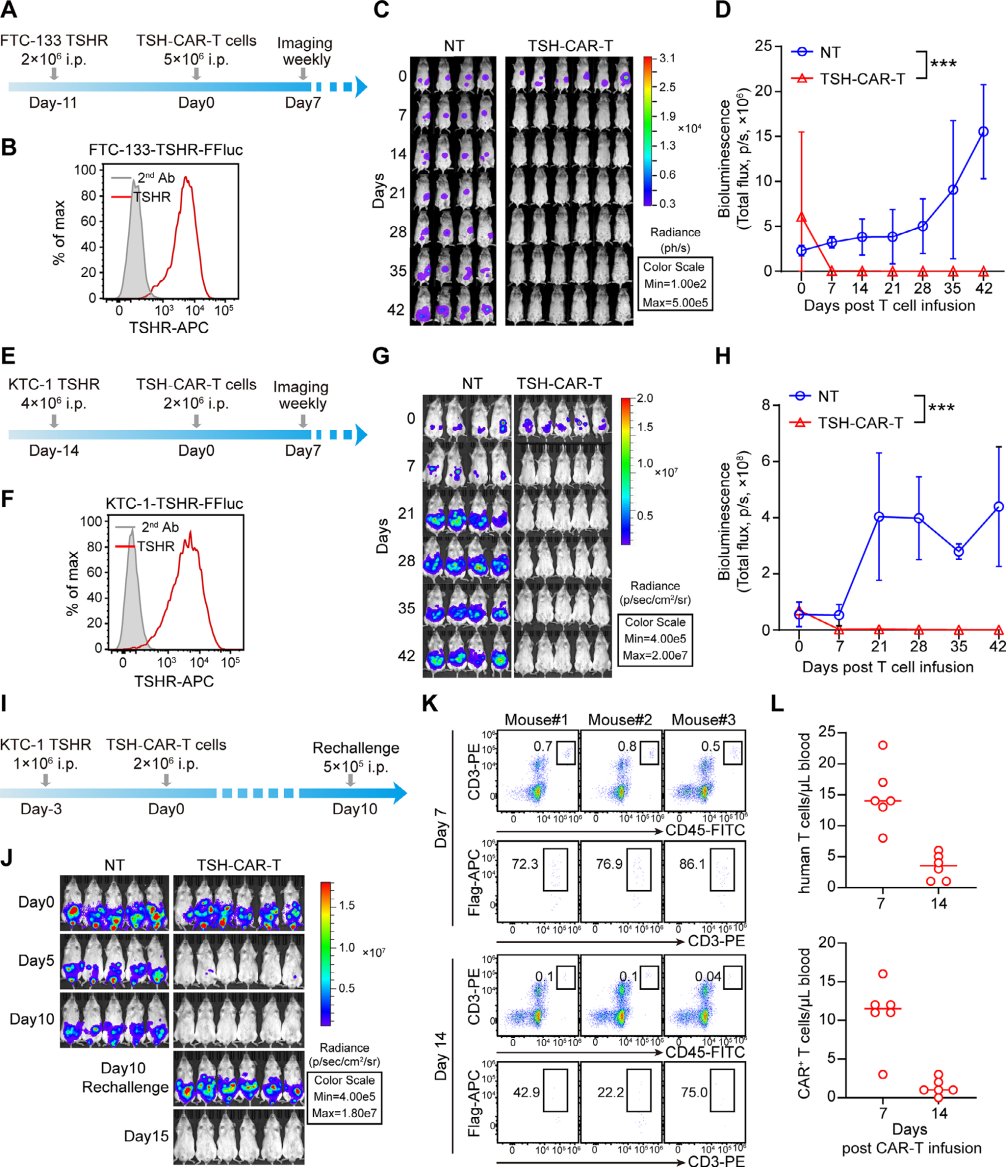
TSH‐CAR‐T effectively controlled tumor growth in xenograft mouse models. A) Schema of the FTC‐133‐TSHR‐FFluc xenograft model. Tumor cells (2 × 10^6^) were injected intraperitoneally into NCG mouse, 11 days later, treated with 5 × 10^6^ NT or CAR‐T cells. B) The expression of TSHR in FTC‐133‐TSHR‐FFluc cell was detected by flow cytometry. C) Representative bioluminescence images of FTC‐133‐TSHR‐FFLuc tumor growth. (NT: *n* = 4; TSH‐CAR‐T: *n* = 6). D) Bioluminescence kinetics of tumor growth in (C). Error bars denote SD. (NT: *n* = 4; TSH‐CAR‐T: *n* = 6). ***p < 0.001; two‐way ANOVA with Tukey's multiple comparisons test correction. E) Schema of the KTC‐1‐TSHR‐FFluc xenograft model. Tumor cells (4 × 10^6^) were injected intraperitoneally into NCG mouse, 14 days later, treated with 2 × 10^6^ NT or CAR‐T cells. F) The expression of TSHR in KTC‐1‐TSHR‐FFluc cell line was detected by flow cytometry. G) Representative bioluminescence images of KTC‐1‐TSHR‐FFLuc tumor growth. (NT: *n* = 4; TSH‐CAR‐T: *n* = 5). H) Bioluminescence kinetics of tumor growth in (G). Error bars denote SD. (NT: *n* = 4; TSH‐CAR‐T: *n* = 5). ***p < 0.001; two‐way ANOVA with Tukey's multiple comparisons test correction. I) Schema of KTC‐1‐TSHR‐FFluc tumor rechallenge model. J) Representative bioluminescence images of the FFluc‐KTC‐1 tumor growth (NT: *n* = 5, TSH‐CAR‐T: *n* = 6). K,L) Representative flow cytometry results showing the CAR‐T cells (CD3^+^CD45^+^Flag^+^) in peripheral blood of the TSH‐CAR‐T cells treated group at days 7 and 14 post CAR‐T cells infusion (K); quantification of total T cells and CAR‐positive T cells is illustrated (L) (*n* = 6).

To evaluate the antitumor effects of TSH‐CAR‐T cells in vivo, we implanted the Firefly luciferase (FFluc) transduced FTC‐133‐TSHR cells into the peritoneal cavity of NCG mice. After 11 days, the mice received an intraperitoneal injection of 5 × 10^6^ NT or TSH‐28ζ‐CAR‐T cells (**Figure** **4**A,B). The tumor growth was monitored by weekly bioluminescence imaging of FFluc expression. The results showed that TSH‐28ζ‐CAR‐T cells effectively controlled FTC‐133 tumor cell growth, maintaining a tumor‐free status for up to 42 days post CAR‐T infusion. Conversely, tumor growth persisted in the NT‐treated cohort (Figure 4C,D).

To further confirm the antitumor efficacy of TSH‐CAR‐T cells, we established another thyroid cancer model in NCG mice using FFluc‐transduced KTC‐1‐TSHR cells. Fourteen days post‐tumor injection, the mice were injected intraperitoneally with 2 × 10^6^ NT or TSH‐CAR‐T cells (Figure 4E,F). In alignment with the results of FTC‐133 tumor model results, TSH‐CAR‐T cells completely eliminated the tumor cells in vivo, and the mice remained tumor‐free for up to 42 days post treatment (Figure 4G,H).

In the tumor rechallenge model, TSH‐CAR‐T cells were capable of completely eradicating the rechallenged tumor cells even 10 days after the initial fully elimination of the originally implanted tumors (Figure 4I,J). Furthermore, the presence of CAR‐T cells in peripheral blood at different time points was analyzed. Despite the CAR‐T cells being administered intraperitoneally, circulating CAR‐T cells were still detectable in the peripheral blood on day 14 following the CAR‐T cell infusion (Figure 4K,L), suggesting that a greater number of CAR‐T cells may be detectable in the peritoneum. Collectively, these findings demonstrate that TSH‐CAR‐T cells possess remarkable antitumor efficacy against TSHR‐positive DTC in vivo.

### 2.5 Murine TSH‐CAR‐T Cells Show Effective Antitumor Activity Against Murine TSHR‐Positive Tumor Cells

**Figure 5:**
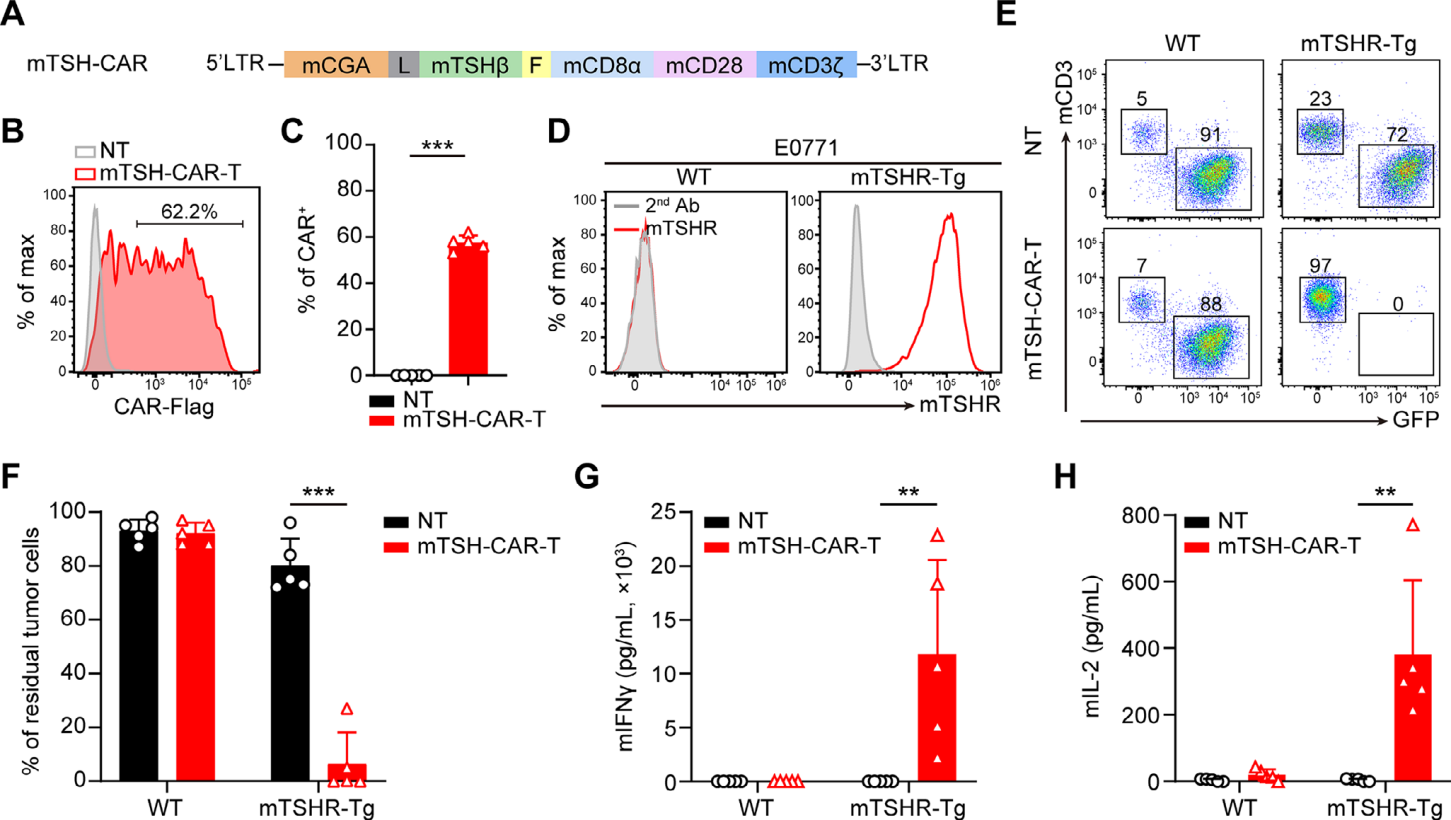
mTSH‐CAR‐T cells specifically eliminated mTSHR‐positive tumor cells. A) Schematic structure of the murine TSH‐CAR construct. mCD8α, the hinge and transmembrane region of murine CD8α; mCD28, intracellular domain of murine CD28; mCD3ζ, intracellular domain of murine CD3ζ; L, (G_4_S)_3_ linker; F, flag tag. B) Representative flow cytometry plots showing the transduction efficiency of mTSH‐CAR. C) Summary of CAR transduction efficiency. Error bars denote SD (*n* = 5). ***p < 0.001; paired *t‐*test with two‐tailed *p*‐value calculation. D) Representative flow plots illustrating mTSHR expression in the wild‐type (WT) and mTSHR exogenously expressed (mTSHR‐Tg) E0771 cell line. E) GFP labeled WT and mTSHR‐Tg E0771 cells were co‐cultured with NT or mTSH‐CAR‐T cells at the T cell to tumor cell ratio of 1–2. On day 5, cancer cells (GFP^+^) and CAR‐T cells (CD3^+^) were enumerated by flow cytometry. F) Summary of residual tumor cells in the co‐culture experiment of (E) (*n* = 5). ***p < 0.001; paired *t‐*test with two‐tailed *p*‐value calculation. G,H) Summary of mIFNγ (G) and mIL‐2 (H) released by CAR‐T cells in the culture supernatant after 24 h of the co‐culture experiment of (E). Error bars denote SD (*n* = 5). **p < 0.01; paired *t‐*test with two‐tailed p value calculation.

For reliably evaluating the safety profile of TSH‐CAR‐T cells using the syngeneic mouse model, we constructed the murine version TSH‐CAR (mTSH‐CAR) for targeting murine TSHR (mTSHR) by linking the murine CGA and TSHβ with a (G_4_S)_3_ linker as the antigen‐binding domain, followed by incorporating murine CD28 costimulatory domain and murine CD3ζ intracellular domain (**Figure** **5**A). Subsequently, the mTSH‐CAR‐T cells were produced by transducing the CAR molecules into the activated T cells of C57BL/6J mice via retrovirus. The expression of CAR was examined by flow cytometry, and the transduction efficiency exceeded 50% (Figure 5B,C).

Due to the absence of mouse thyroid cancer cell lines, a mouse breast cancer cell line E0771 was selected, and subsequently, the mTSHR was exogenously expressed into the E0771 cells (E0771‐mTSHR), creating a mTSHR‐positive target cell (Figure 5D). To evaluate the specificity and effectiveness of mTSH‐CAR‐T cells against mTSHR‐positive tumor cells, the E0771 wild type (E0771‐WT) and E0771‐mTSHR tumor cells were co‐cultured with NT and mTSH‐CAR‐T cells at an effector‐to‐target ratio (E:T) of 1:2 for 5 days. The results showed that mTSH‐CAR‐T cells specifically targeted and eliminated the E0771‐mTSHR tumor cells while sparing the E0771‐WT tumor cells (Figure 5E,F). No cytotoxic effects were observed in the NT‐treated group (Figure 5E,F). The cytolytic activity of mTSH‐CAR‐T cells was further validated by the significant release of cytokines (mIFNγ and mIL‐2) into the supernatant when co‐cultured with E0771‐mTSHR, which was not observed in the supernatant from co‐cultured with E0771‐WT (Figure 5G,H). These findings demonstrated the specific killing capability of mTSH‐CAR‐T cells toward mTSHR‐positive tumor cells.

### 2.6 mTSH‐CAR‐T Cells Exhibit a Favorable Safety Profile with Restricted Transient and Reversible Impairment to Thyroid Follicles

**Figure 6:**
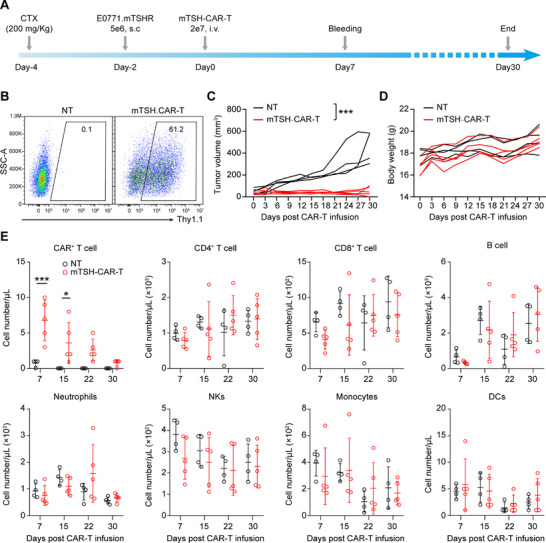
mTSH‐CAR‐T cells effectively control tumor growth in immunocompetent mice without depleting peripheral blood cells. A) Schema of the safety evaluation experiment of mTSH‐CAR‐T cells in immunocompetent syngeneic mouse model. C57BL/6J mice received a pretreatment of CTX (200 mg kg^−1^) 2 days before the subcutaneous injection of E0771‐mTSHR cells (5 × 10^6^), followed by an intravenous administration of 2 × 10^7^ mTSH‐CAR‐T cells 2 days later. B) Representative flow cytometry plots showing the expression of CARs in the CAR‐T cells. C,D) The tumor volume (C) and body weight (D) were measured every 3 days after CAR‐T cells infusion (NT: *n* = 4; mTSH‐CAR‐T: *n* = 5). ***p < 0.001; two‐way ANOVA with Tukey's multiple comparisons test correction. E) The immune cell composition of the blood was analyzed by flow cytometry at day 7, 15, 22, and 30 after CAR‐T cell infusion. *p < 0.05 and ***p < 0.001; unpaired and non‐parametric Mann–Whitney test with two‐tailed *p*‐value calculation.

To comprehensively investigate the safety profile of mTSH‐CAR‐T cells, we established a syngeneic tumor model in immunocompetent C57BL/6J mice. Six‐week‐old C57BL/6J mice were treated with cyclophosphamide (CTX, 200 mg Kg^−1^) to create a lymphodepletion microenvironment favorable for the expansion of CAR‐T cells. Two days afterward, E0771‐mTSHR tumor cells were subcutaneously implanted into the mice, and syngeneic NT and mTSH‐CAR‐T cells were administered intravenously 2 days post‐tumor implantation (**Figure** **6**A). The results showed that mTSH‐CAR‐T cells nearly entirely eliminated the tumors (Figure 6B,C). In addition, the body weight remained stable in the mTSH‐CAR‐T cells‐treated group, suggesting no lethal toxicity associated with mTSH‐CAR‐T cells (Figure 6D).

**Figure 7:**
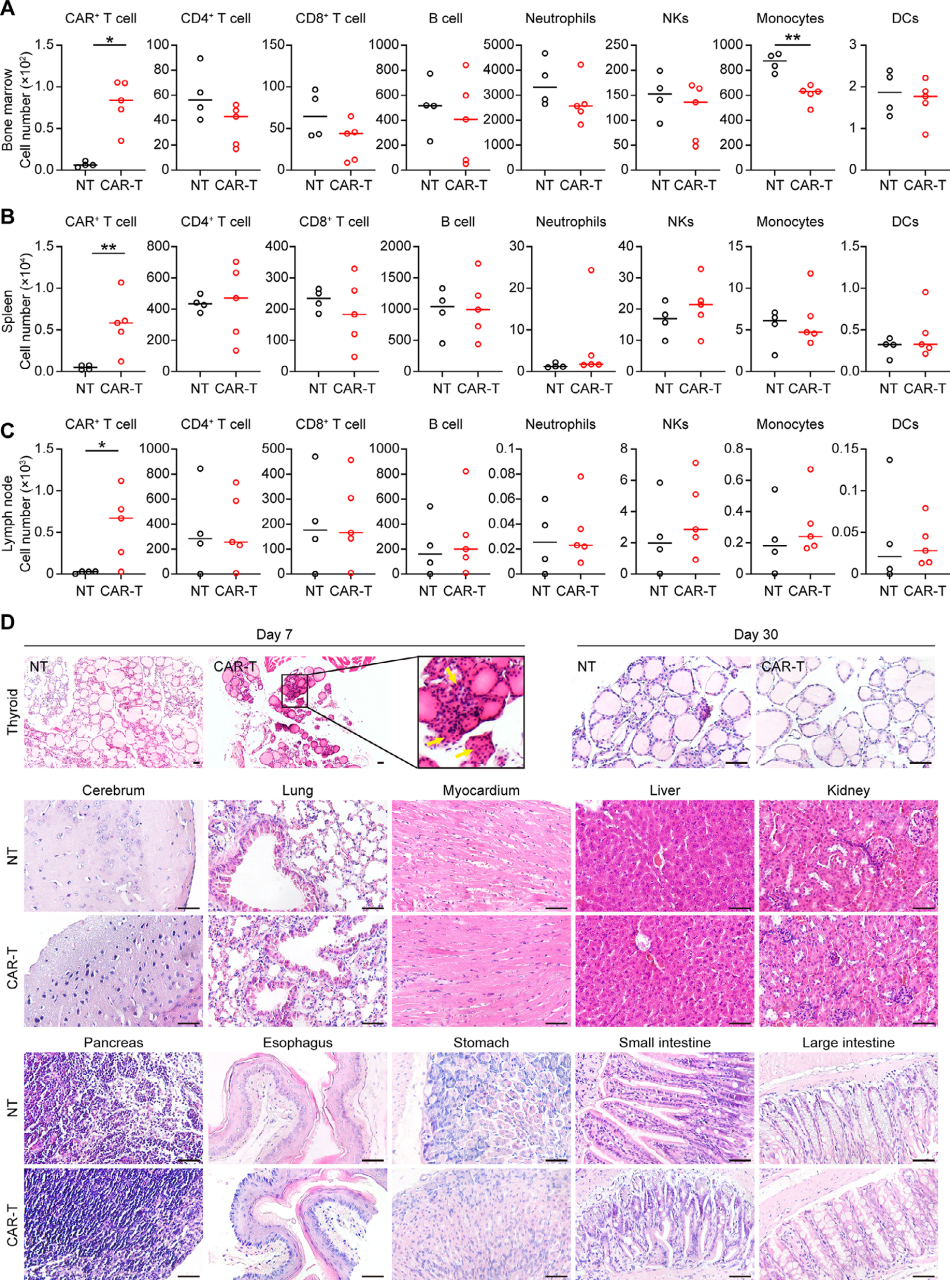
mTSH‐CAR‐T cells do not exhibit on target/off tumor effect except transient impairment to thyroid follicles. A–C) The mice from the safety evaluation model were euthanized 30 days post CAR‐T cell infusion, the immune cell composition of the bone marrow (A), spleen (B), and lymph node (C) was analyzed by flow cytometry. *p < 0.05 and **p < 0.01; unpaired and non‐parametric Mann–Whitney test with two‐tailed *p*‐value calculation. D) The tissue pathology of thyroid was examined on day 7 and 30 post mTSH‐CAR‐T infusion. The tissue pathology of other organs was examined on day 30 (NT: *n* = 4; mTSH‐CAR‐T: *n* = 4). Scale bars are 100 µm.

Furthermore, peripheral blood was collected on day 7, 15, 22, and 30 following CAR‐T cell infusion, and flow cytometry was employed to analyze various immune cell types, including CAR^+^ T cells, CD4^+^ and CD8^+^ T cells, B cells, natural killer cells (NKs), monocytes, neutrophils, and dendritic cells (DCs). The results revealed that the CAR^+^ T cells were detectable in the peripheral blood of mTSH‐CAR‐T treated mice from day 7 through day 22 post CAR‐T cells infusion (Figure 6E; Figure S6, Supporting Information), with no significant difference in the various immune cell types assessed between NT and mTSH‐CAR‐T cells treated mice (Figure 6E). Similarly, at the endpoint of the experiment, mice treated with mTSH‐CAR‐T cells exhibited no reduction of any immune cell types in bone marrow, spleen and lymph nodes (**Figure** **7**A–C). These findings demonstrate that mTSH‐CAR‐T cells do not exert off‐target effects on immune cells.

Additionally, to examine whether mTSH‐CAR‐T cells impacted any normal organs, various organs were harvested, and HE staining was performed to check for any potential tissue damage or abnormal infiltration of CAR‐T cells. The results showed that there is no observable tissue damage in the mTSH‐CAR‐T cells‐treated mice compared to the NT cells‐treated mice, except the impaired thyroid follicles and increased lymphocyte infiltration were observed on day 7 post‐mTSH‐CAR‐T cells treatment (Figure 7D; Figures S7 and S8, Supporting Information). However, no damage to the thyroid follicles was detectable 30 days after mTSH‐CAR‐T cells treatment. This absence of impairment may be attributed to the significant reduction of CAR‐T cells 30 days after infusion, allowing for self‐repair of the harmed thyroid follicles, rendering them undetectable by day 30. Overall, these findings demonstrate that mTSH‐CAR‐T cells effectively control the tumor growth in immunocompetent C57BL/6J mice without causing detectable organ toxicity, aside from the thyroid tissues.

Furthermore, the anti‐tumor efficacy of mTSH‐CAR‐T cells was also verified in an alternative B16‐mTSHR syngeneic tumor model. mTSH‐CAR‐T cells significantly inhibited tumor growth and prolonged the survival of the mice (Figure S9A–F, Supporting Information). To determine if mTSH‐CAR‐T cells would cooperate with endogenous T cells or reshape the tumor microenvironment to achieve anti‐tumor effects, we examined the activation of both endogenous and exogenous T cells in peripheral blood, nearby lymph nodes and tumor tissues, as well as the proportion of other immune cell types within tumor tissues, discovering that the exogenous mTSH‐CAR‐T exhibited apparently activated phenotype in both the tumor and adjacent lymph nodes, characterized by significantly elevated expression level of CD69 and PD1, in contrast, there was no significant difference in endogenous T cells between NT and mTSH‐CAR‐T treated groups (Figure S9G–I, Supporting Information). Furthermore, no considerable differences were noted in the proportions of other immune cell types within the tumor microenvironment (Figure S9J, Supporting Information). These findings indicated that mTSH‐CAR‐T cells were primarily responsible for the anti‐tumor effect, rather than the endogenous T cells or alterations in the tumor immune environment.

## 3 Discussion

This study presents the development and preclinical validation of a ligand‐based CAR‐T cell therapy targeting TSHR for the treatment of DTC. Our findings demonstrated that TSHR represents an exceptional tumor‐specific antigen for CAR‐T cell therapy in DTC, with high and consistent expression in primary and conventional treatment‐resistant tumors while being entirely absent in normal tissues outside the thyroid.^[^ ^29^, ^31^, ^32^ ^]^ Critically, the surgical removal of thyroid tissue in thyroid cancer treatment uniquely eliminates the risk of on‐target/off‐tumor toxicity against healthy TSHR‐expressing cells, a major limitation plaguing CAR‐T therapy for many other solid tumors.

Beyond target selection, we addressed a second fundamental barrier to the effectiveness of CAR‐T therapy in solid tumors, which is immunogenicity. Conventional scFv‐based CARs, typically derived from murine monoclonal antibodies (mAbs), can provoke host immune rejection, limiting CAR‐T persistence and long‐term antitumor activity.^[^ ^35^, ^42^ ^]^ Even humanized mAbs cannot fully avoid immunogenicity because they don't belong to the housekeeping gene‐encoded protein.^[^ ^43^ ^]^ Our approach utilizes TSH, the natural ligand of TSHR, as the antigen‐binding domain. As a human “housekeeping” protein, TSH is inherently non‐immunogenic, bypassing the risk of anti‐CAR immune response.^[^ ^44^ ^]^

Based on this principle, we have successfully engineered functional TSH‐CAR constructs that incorporate either CD28 or 4‐1BB costimulatory domains. Both variants displayed robust and specific cytotoxicity against TSHR‐expressing DTC cell lines in vitro, accompanied by potent cytokine release (IFNγ and IL‐2) and T‐cell proliferation upon antigen engagement. However, CD28‐expressing TSH‐CAR‐T cells released higher levels of IL‐2 and IFNr compared to those carrying 4‐1BB, which aligns with existing findings that the CD28 costimulatory molecule induces faster and stronger activation of CAR‐T cells.^[^ ^45^, ^46^ ^]^ Importantly, the anti‐tumor activity of TSH‐CAR‐T cells was comparable to that of control CAR‐T cells that target the pan‐tumor antigen B7‐H3. Notably, the orientation of the TSH subunits (CGA‐TSHβ) was crucial for functionality, as the reverse construct (TSHβ‐CGA) showed impaired effectiveness. In vivo efficacy was unequivocally demonstrated in two distinct xenogeneic mouse models, where TSH‐CAR‐T cells accomplished complete tumor eradication and sustained remission.

Although our IHC results, along with those from another research group, indicate that TSHR protein is exclusively expressed in the thyroid,^[^ ^32^ ^]^ existing studies have shown that TSHR is also expressed in normal tissues other than the thyroid, including adipose tissue and kidney.^[^ ^24^, ^47^, ^48^, ^49^ ^]^ Therefore, it is necessary to comprehensively examine the safety profile of CAR‐T cells targeting TSHR in a reliable mouse model. The traditional scFv‐based CAR targeted at TSHR could not accomplish this purpose in mouse models,^[^ ^32^ ^]^ as it fails to recognize murine antigen, and assessing safety directly in patients could lead to potentially life‐threatening toxicities if the safety profile remains unclear.^[^ ^50^, ^51^ ^]^

A pivotal strength of this ligand‐based CAR design is its inherent translatability to syngeneic models for rigorous safety assessment. By constructing a murine TSH‐CAR (mTSH‐CAR), we can accurately assess the safety profile in immunocompetent C57BL/6J mice. mTSH‐CAR‐T cells demonstrated potent anti‐tumor activity against mTSHR‐expressing tumors while exhibiting a favorable safety profile. Crucially, comprehensive immune cell profiling and histopathological examination of major organs revealed no evidence of off‐target toxicity or damage outside the thyroid. The observed transient impairment of thyroid follicles, consistent with the anticipated on‐target effect, was reversible and resolved by day 30, coinciding with the contraction of CAR‐T cells. This transient side effect is deemed clinically acceptable, especially following the prior removal of the thyroid in DTC patients. The absence of toxicity elsewhere emphasizes the exceptional specificity of this ligand‐receptor system.

Our study offers compelling evidence that TSH ligand‐based CAR‐T therapy targeting TSHR is a highly promising approach for advanced DTC. The combination of a truly tumor‐restricted antigen (post‐thyroidectomy) alongside the non‐immunogenic CAR design utilizing the natural ligand tackles two major hurdles in solid tumor CAR‐T therapies. The potent efficacy seen in xenograft models, coupled with the positive safety profile demonstrated in the stringent syngeneic model, strongly supports the clinical translation of this approach. It holds particular significance for patients with metastatic or radioactive iodine‐resistant DTC, who currently confront poor prognoses and very limited therapeutic alternatives.

Even though the findings are incredibly promising, there are several directions worth further exploring in the future. First, due to the impact of epigenetic alterations^[^ ^52^ ^]^ and other unknown reasons, DTC cell lines have lost TSHR expression in vitro. As a result, some experiments in this study relied on exogenously expressed TSHR, which may exceed the levels of endogenously expressed TSHR in tumor cells from patients. Thus, it is important to further validate the anti‐tumor efficacy of TSH‐CAR‐T cells in patient‐derived tumor xenograft models in the future. Second, the absence of on‐target/off‐tumor effects and the non‐immunogenic CAR design allow for the potential of further enhancing antitumor activity and long‐term persistence, such as combine with controllable co‐expression of cytokines and cytokine receptors, such as constitutively activated IL7 receptors.^[^ ^53^, ^54^ ^]^ Third, exploring the combination with agents modulating the tumor microenvironment or enhancing CAR‐T cell trafficking, could further refine therapeutic outcomes,^[^ ^55^, ^56^ ^]^ as well as the potential of combination with tyrosine kinase inhibitors that are currently used in advanced thyroid cancer,^[^ ^57^, ^58^ ^]^ to improve the effectiveness of TSH‐CAR‐T cell therapy. Lastly, the transient thyroid effects noted in the murine model, while reversible and of minor clinical significance, suggests it is necessary to monitor thyroid hormone levels in forthcoming clinical trials involving patients with residual thyroid tissue (post‐partial thyroidectomy), although this is not anticipated to be a major clinical concern.

In conclusion, we have successfully developed an innovative, ligand‐based TSH‐CAR‐T cell therapy targeting TSHR for DTC. This approach leverages the exceptional tumor specificity of TSHR in thyroidectomized DTC patients and overcomes the immunogenicity barrier by employing the natural ligand. The robust preclinical effectiveness and exceptionally favorable safety profile strongly support the clinical development of TSH‐CAR‐T as a promising new therapeutic option for patients with advanced, conventional treatment‐resistant DTC.

## 4 Experimental Section

### Cell Lines and Cell Culture

KTC‐1 (RRID: CVCL_6300), B16 (RRID: CVCL_F936), and E0771 (RRID: CVCL_GR23) cell lines were purchased from the National Collection of Authenticated Cell Cultures, Shanghai. FTC‐133 (RRID: CVCL_1219) and 293T (RRID: CVCL_0063) cell lines were purchased from American Type Culture Collection (ATCC). TPC‐1 (RRID: CVCL_6298) cell line was purchased from Shanghai Zhong Qiao Xin Zhou Biotechnology Co.,Ltd. TPC‐1, KTC‐1, and FTC‐133 cell lines were cultured in RPMI‐1640 medium (Gibco) supplemented with 10% Fetal bovine serum (FBS, Sunrise), 2 mm GlutaMax, 100 unit mL^−1^ of Penicillin and 100 mg mL^−1^ of streptomycin (100 unit mL^−1^ of P/S). B16 and E0771 cell line was cultured in Dulbecco's Modified Eagle's Medium (DMEM, Gibco) supplemented with 10% FBS (Sunrise), 2 mm GlutaMax and 100 unit mL^−1^ of P/S. 293T (RRID: CVCL_KS62) cell line was cultured in Iscove's Modified Dulbecco Medium (IMDM, Gibco) supplemented with 10% FBS (Gibco), 2 mm GlutaMax and 100 unit mL^−1^ of P/S. All cells were maintained in a humidified atmosphere (Thermo Fisher Scientific) containing 5% CO_2_ at 37 °C. All cell lines were mycoplasma‐free, and tested regularly during the experiment.

### Human and Mouse Tissue Samples

Human thyroid tumor tissue microarray and human normal tissue microarray were purchased from Shanghai OUTDO BioTech. Mouse tissues were obtained from the C57BL/6J mice at the endpoint of the experiment.

### Plasmid Construction

The full length of human TSHR genes were amplified by PCR from cDNA of breast cancer cell line SKOV3, and cloned into the lentiviral vector pCDH‐IRES‐GFP. Murine TSHR gene was amplified by PCR from cDNA of mouse subcutaneous fat pad, and cloned into the lentiviral vector pCDH‐IRES‐GFP. The mTSH‐Fc and hTSH‐Fc chimera protein were generated by fusing the coding region of murine or human TSHα and TSHβ subunits with the human IgG1‐Fc region by PCR, and then cloned into the lentiviral vector pCDH‐IRES‐GFP.

The human TSHα and TSHβ genes were synthesized by GENEWIZ from Azenta Life Science, and cloned into the previously validated CAR formats to replace scFv fragment as the antigen‐binding domain, followed by the human CD8α hinge and transmembrane domain, CD28 or 4‐1BB intracellular costimulatory domains and the intracellular CD3ζ signaling domain. The TSH‐CAR cassettes were cloned into the retroviral vector SFG, and a Flag tag sequence was inserted between the TSH and CD8α hinge sequences to indicate the expression of CAR. For the murine mTSH‐CAR, the murine TSHα and TSHβ genes were amplified by PCR from the cDNA of mouse thymus tissues, and the human CD8α, CD28, and CD3ζ sequences were replaced by the corresponding murine CD8α, CD28, and CD3ζ sequences. The thy1.1 was added following CD3ζ sequence for detecting the CAR expression of mTSH‐CAR‐T cells. The CD19‐CAR and B7‐H3‐CAR used in this study were previously reported.^[^ ^59^ ^]^

### Virus Preparation

Retrovirus used for the transduction of human T cells was prepared as previously reported.^[^ ^59^ ^]^ Briefly, 2 × 10^6^ 293T cells were plated into the 10 cm cell culture dish and transfected with the plasmid mixture of retroviral vector, RDF and Peg‐Pam plasmid using the GeneJuice transfection reagent (Merck Millipore), according to the manufacturer's instruction. Retrovirus used for the transduction of murine T cells was generated by the transfecting 293T cells with the indicated retroviral vector and pCL‐Eco plasmid. The supernatant containing the retrovirus was collected 48 and 72 h after transfection, and filtered by 0.45 µm filters.

The lentivirus preparation process for transduction of human thyroid cancer cell lines and mouse cancer cell line was the same. Briefly, 4 × 10^6^ 293T cells were plated into the 10 cm cell culture dish and transfected with the plasmid mixture of lentiviral vector, pMD2.G and PsPAX2 plasmid using the GeneJuice transfection reagent, according to the manufacturer's instruction. The supernatant containing the lentivirus was collected 48 h after transfection, and filtered by 0.45 µm filters.

### Cell Line Construction

Thyroid cancer cell lines TPC‐1, KTC‐1, and FTC‐133 and murine cancer cell line E0771 were transduced with lentiviral vector encoding GFP and human or murine TSHR and GFP for in vitro coculture experiment. For in vivo experiment, KTC‐1 and FTC‐133 cell lines were transduced with lentiviral vector encoding human TSHR and Firefly‐luciferase (FFluc). B16 and E0771 cells were transduced with lentiviral vector encoding mTSHR.

### Preparation of mTSH‐Fc and hTSH‐Fc Chimera Protein for mTSHR and hTSHR Staining

The stable 293T cell line expressing mTSH‐Fc or hTSH‐Fc chimera protein was established by lentiviral transduction. The cells were then seeded into the six‐well plate and the supernatant was collected at 48 h, filtered with 0.22 µm filter and used for staining of the mTSHR on E0771 cells and hTSHR on T cells and human cancer cell lines.

### Generation of Human CAR‐T Cells

Peripheral blood mononuclear cells (PBMCs) were isolated by density gradient separation from healthy volunteer blood recruited at the Affiliated Hospital of Xuzhou Medical University. The study was approved by the Ethics Committee of the Affiliated Hospital of Xuzhou Medical University, and informed consent was obtained from all volunteers included in this study. The PBMCs were activated by anti‐CD3 (1 µg mL^−1^) and anti‐CD28 (1 µg mL^−1^) antibody. After 2 days, the activated T cells were transduced with retrovirus using RetroNectin‐coated plate according to the reported procedure. Briefly, non‐tissue culture‐treated plate were coated with 7 µg mL^−1^ RetroNectin overnight at 4 °C. The plate was washed once with 1 mL medium, and then 1 mL retrovirus was added and centrifuged at 2000 g for 90 min. Supernatant was discarded, and 3.5 × 10^5^ activated T cells were added into the plate, and centrifuged at 1000 g for 10 min. T cells were expanded in the RPMI‐1640 medium supplemented with 10% FBS (Hyclone), 2 mM GlutaMax, 100 unit mL^−1^ of P/S, IL7 (10 ng mL^−1^), and IL15 (5 ng mL^−1^). On day 10–12, cells were collected for in vitro and in vivo experiments.

### Generation of Murine CAR‐T Cells

T cells were isolated from murine spleen using the Mouse CD3 T cell isolation Kit (BioLegend) according to the manufacturer's instruction. T cells were activated on plates coated with 1 µg mL^−1^ mCD3 and 1 µg mL^−1^ mCD28 antibody for 24 h. Activated T cells were transduced by retrovirus using the same protocol as human CAR‐T preparation. T cells were expanded in complete medium (RPMI‐1640 (Sigma), 10% FBS (Gibco), 2 mM GlutaMax, 100 unit mL^−1^ of P/S, 75 µm β‐mercaptoethanol) with IL7 (10 ng mL^−1^) and IL15 (5 ng mL^−1^). On day 5 after transduction, T cells were collected for in vitro and in vivo experiments.

### Coculture Experiment

Tumor cells were seeded in 24‐well plate at a concentration of 2.5 × 10^5^ cells per well. After the tumor cells were attached to the plate, T cells were added at an effector‐to‐target ratio of 1:5. Cells were collected to measure the residual tumor and T cells at day 5. Dead cells were gated out by Zombie Aqua^TM^ Fixable Viability Kit (BioLegend) staining, while T cells and tumor cells were marked by anti‐CD3 antibody and GFP, respectively.

To evaluate the effect of free TSH on the cytotoxic activity of TSH‐CAR‐T cells, the concentration of TSH‐Fc protein in the supernatant of 293T cells expressing TSH‐Fc protein was measured by Human IgG Fc Fragment ELISA Kit (Mabtech, 3850‐1H‐6). Different concentrations of TSH‐Fc protein were added to the above‐mentioned co‐culture system, and cells were collected to measure the residual tumor and T cells at day 5.

### ELISA

T cells were cocultured with tumor cell at an effector‐to‐target ratio of 1:5. After 24 h, the supernatant was collected and cytokines (IFNγ and IL‐2) were measured using indicated ELISA kits according to the manufacturer's instruction.

### CFSE Dilution Assay

T cells were stained with carboxyfluorescein diacetate succinimidyl ester (CFSE, 1.5 mm) and cocultured with tumor cells at a ratio of 1:1. On day 5, T cells were gated based on CD3 expression and the fluorescence intensity of CFSE was analyzed by flow cytometry.

### Flow Cytometry

Flow cytometry was performed using the following antibodies: Human cells were stained with anti‐CD3, anti‐CD4, anti‐CD8, anti‐CD45RA, and anti‐CD62L antibodies conjugated with PE, FITC, and APC‐Cy7 fluorochromes. The expression of TSHR on thyroid cancer lines and T cells was detected by the anti‐TSHR monoclonal antibody (Abcam, ab218108), followed by APC‐conjugated Goat anti‐rabbit IgG secondary antibody (BD Biosciences). Murine cells were stained with anti‐CD4, anti‐CD8, anti‐CD3, anti‐CD45, anti‐CD49b, anti‐CD11b, anti‐B220, anti‐Ly6G and anti‐CD11c antibodies conjugated with FITC, APC, PE, BV421, PE‐Cy5, FITC and Percp fluorochromes. Expression of B7‐H3 on the thyroid cancer cell lines was assessed with the B7‐H3 mAb clone EPNCIR122 (Abcam, ab134161) combined with the Alexa Fluor 647 Donkey anti‐rabbit IgG (minimal x‐reactivity) Antibody (BioLegend, 406414). The expression of murine TSHR on E0771 and B16 cell lines was detected using mTSH‐Fc chimera protein followed by the Alexa Fluor 647 Goat anti‐human IgG antibody (Jackson ImmunoResearch Laboratories INC, 109‐606‐088). The TSH‐CAR expression on human and murine T cells was detected by the anti‐Flag or anti‐Thy1.1 antibodies conjugated with APC and FITC fluorochromes, respectively. Expression of B7‐H3‐CAR and CD19‐CAR was detected using B7‐H3‐Fc chimera protein (Sino Biological, 11188‐H02H), followed by the Alexa Fluor 647 Goat anti‐human IgG antibody and the anti‐idiotype mAb (clone 233‐4A),^[^ ^60^ ^]^ respectively.

### Immunohistochemistry and Tissue Histopathology

A tissue microarray containing 23 different types of normal human tissues (OD‐NH‐Com01‐001) and human thyroid cancer tissue microarray (including 28 cases of cancer tissue and adjacent normal thyroid tissue) (HThy‐Can060PT‐01) were purchased from Shanghai Outdo Biotech Co., Ltd. The section was probed with primary antibody against TSHR (Ab218108, 1:1000) at 4 °C overnight and incubated with the Goat anti‐Rabbit secondary antibody at room temperature for 1 h, followed by color development 3,3′‐diaminobenzidine chromogen. Hematoxylin counterstain was used for nuclei visualization.

### Xenogeneic Mouse Models

All the mouse experiments were performed in accordance with the Xuzhou Medical University animal husbandry guidelines according to protocols approved by the Animal Care and Use Committee of Xuzhou Medical University. Four‐ to six‐week‐old female NCG mice were purchased from GemPharmatech and housed in a specific pathogen‐free (SPF) animal facility of Xuzhou Medical University. Mice were maintained under specific‐pathogen‐free conditions with daily cycles of 12‐h light and 12‐h darkness, and health monitoring was carried out on a regular basis. Animals were euthanized upon showing symptoms of clinically overt disease (not feeding, lack of activity, abnormal grooming behavior, hunched back posture) or excessive weight loss (15% body‐weight loss over 1 week). The animal study was approved by the Ethics Committee of Xuzhou Medical University (202109A168).

For the FFluc‐FTC‐133 model, 2 × 10^6^ tumor cells suspended in matrigel were inoculated intraperitoneally into an 8‐week‐old female NCG mouse. Eleven days post tumor inoculation, 5 × 10^6^ NT or TSH‐CAR‐T cells were injected intraperitoneally. For the KTC‐1 tumor model, 4 × 10^6^ tumor cells suspended in matrigel were inoculated intraperitoneally into an 8‐week‐old female NCG mouse. After 14 days, 2 × 10^6^ NT or TSH‐CAR‐T cells were infused by intraperitoneally. In each experiment, the mice were grouped randomly based on the bioluminescence imaging using Berthold NightOWL II LB983 or IVIS Spectrum in vivo imaging system. The growth of tumor cells was monitored weekly using the bioluminescence system.

For the KTC‐1‐TSHR re‐challenge model, 6‐week‐old male mice were injected intraperitoneally with KTC‐1‐TSHR‐FFluc tumor cells (1 × 10^6^ cells per mouse). On day 3 after tumor cell inoculation, NT or TSH‐CAR‐T cells were injected intraperitoneally (2 ×10^6^ cells per mouse). Ten days after CAR‐T cell treatment, mice were re‐challenged with 5 × 10^5^ KTC‐1‐TSHR‐FFluc tumor cells intraperitoneally.

### Safety Evaluation of TSH‐CAR‐T Cells in the Syngeneic Mouse Model

For E0771 tumor model, 6‐week‐old female C57BL/6J mice were pre‐treated with 200 mg Kg^−1^ of cyclophosphamide (CTX) to create a lymphodepleted environment. Two days later, 5 × 10^6^ E0771‐mTSHR tumor cells were inoculated subcutaneously. Two days post‐tumor cell implanted, 2 × 10^7^ NT or mTSH‐CAR‐T cells were injected intravenously. The tumor volume was measured every 3 days. The cell composition of peripheral blood was assessed by flow cytometry and counting beads weekly. Mice were euthanized 30 days post T cell infusion. Immune cell composition of blood, spleen, lymph node, and bone marrow were assessed by flow cytometry and counting beads. The different types of organs were collected and potential tissue damage was examined by hematoxylin and eosin staining. In the parallel experiments, mice were sacrificed on day 7 after CAR‐T infusion to collect thyroid and other normal tissues for detection of tissue damage by HE staining and CAR‐T cell infiltration by flow cytometry.

For the B16 tumor model, 6‐week‐old female C57BL/6J mice were pre‐treated with 200 mg Kg^−1^ of cyclophosphamide (CTX) to create a lymphodepleted environment. Two days later, 5 × 10^5^ B16‐mTSHR tumor cells were inoculated subcutaneously. Fourteen days post‐tumor cell implanted, 1 × 10^7^ NT or mTSH‐CAR‐T cells were injected intravenously. The tumor volume was measured every 2 days. In the parallel experiments, 1 × 10^6^ B16‐mTSHR tumor cells were inoculated subcutaneously. Seven days post‐tumor cell implanted, 1 × 10^7^ NT or mTSH‐CAR‐T cells were injected intravenously, and mice were sacrificed on day 5 after CAR‐T infusion to detect the types of immune cells and the activation of CAR‐T cells in tumor tissues.

### Statistical Analysis

All experiments were repeated at least three times, and the experimental data were analyzed by Graphad Prism software. The data were shown as mean ± SD. The paired or unpaired and nonparametric Mann–Whitney test with two‐tailed *p*‐value calculation was used to measure the difference between two groups. The one‐way ANOVA was used to determine statistically differences between multiple groups. The statistical analysis method is also described in the figure legend. The *p*‐value < 0.05 indicates a significant difference.

## Conflict of Interest

H.W.D., F.W., H.Z., and J.N.Z. filed a patent for the TSH‐CAR targeting TSHR, the patent number is ZL202310076013.6.

## Author Contributions

F.W. and H.Z. contributed equally to this work. H.W.D., C.X., J.N.Z., and G.W. designed and supervised the study. F.W. and H.Z. conducted the in vitro and in vivo experiments. L.L., S.Y.W., and H.F.Z. provided assistance for in vitro experiments and mouse experiments. Z.L. provided assistance for the IHC experiments on human samples. Writing of the original draft was carried out by F.W., H.Z., and H.W.D. Review and editing were conducted by all authors.

## Supporting information



Supporting Information

## Data Availability

The data that support the findings of this study are available in the supplementary material of this article.
