# Supplementary material for: TSH Ligand‐Based CAR‐T Cell Effectively Eradicates TSHR‐Positive Thyroid Cancer with Favorable Safety Profile
Source: Adv Sci (Weinh). 2025 Sep 23;12(46):e13243. doi: 10.1002/advs.202513243 (PMC12697831; doi:10.1002/advs.202513243)
Supplement: Supplementary file 1 — Supporting Information [file ADVS-12-e13243-s001.pdf]

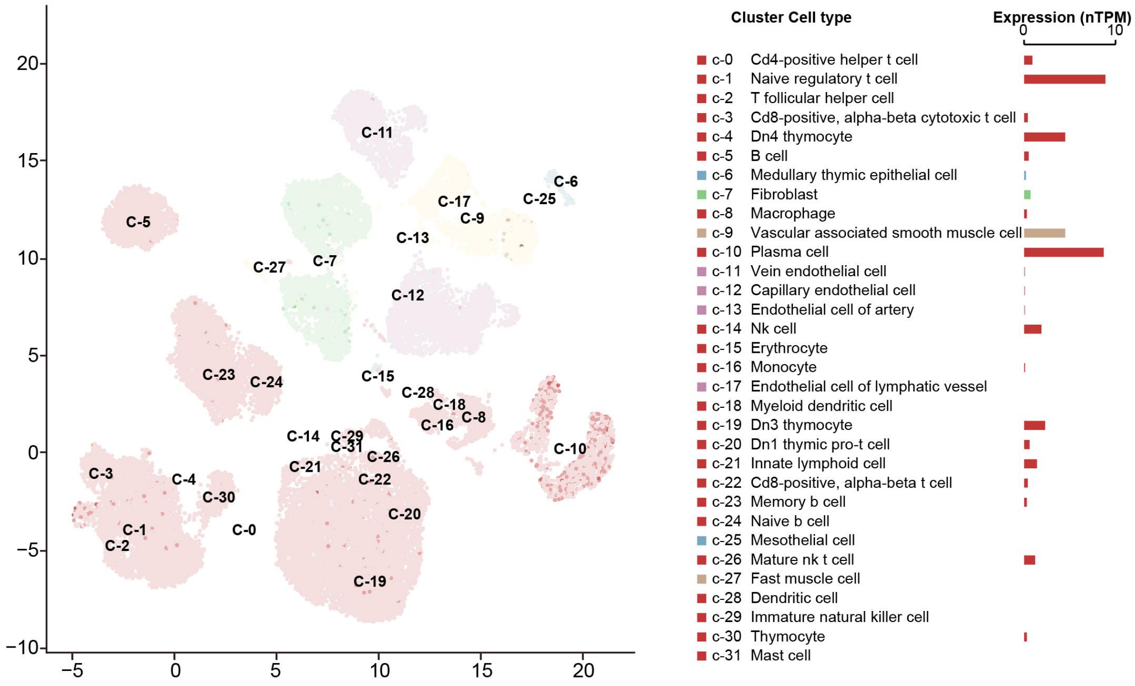

Figure S1. The mRNA of TSHR is sporadically and barely present in a limited proportion of plasma cells within thymus. Single-cell sequencing data from Protein Atlas was used to analyze the mRNA level of TSHR in various cell types of thymus (https://www.proteinatlas.org/).

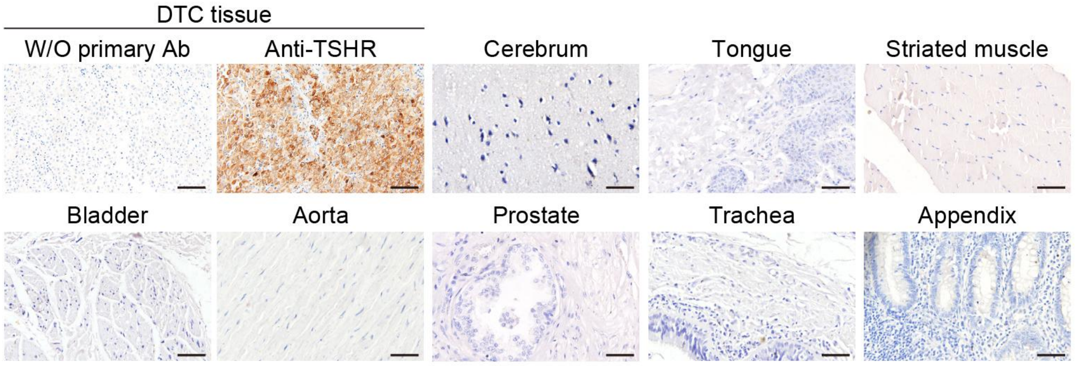

Figure S2. TSHR is not expressed in the indicated normal human tissues. The expression of TSHR in the indicated normal human tissues was examined using anti-TSHR mAb EPR19751 via IHC. DTC tissue sections were used as positive control, W/O primary Ab is negative control. Scale bars are 100 μm.

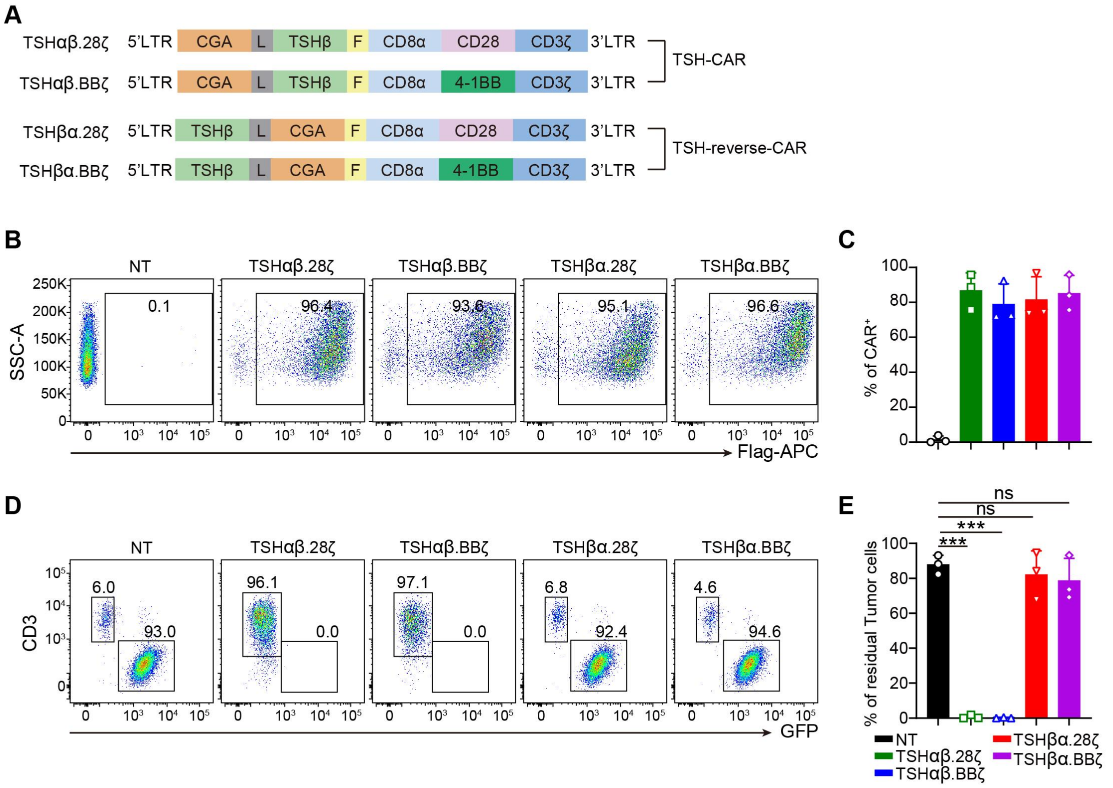

Figure S3. TSH-reverse-CAR-T cells have no killing function.

(A) Schematic structure of the TSH-CAR and TSH-reverse-CAR. CD8α, the hinge and transmembrane region of CD8α; CD28, intracellular domain of CD28; 4-1BB, intracellular domain of 4-1BB; CD3ζ, intracellular domain of CD3ζ; L, $(G_4S)_3$ linker; F, flag tag.

(B) Representative flow cytometry plots showing expression of CARs in CAR-T cells via staining the flag tag.

(C) Summary of the CARs transduction efficiency. Data were shown as individual values and the mean ± SD (n= 3).

(D-E) FTC133-TSHR cancer cells labeled with GFP were co-cultured with NT, TSH-CAR-Ts, or TSH-reverse-CAR-Ts at the T cell to tumor cell ratio of 1 to 5. On day 5, cancer cells (GFP^+) and CAR-T cells ($CD3^+$) were enumerated by flow cytometry. Representative flow-cytometry plots (D) and quantification of residual tumor cells (E) are illustrated (n = 3). Error bars denote SD. ****P* < 0.001; ns, not significant; two-way ANOVA with Tukey's multiple comparisons test correction.

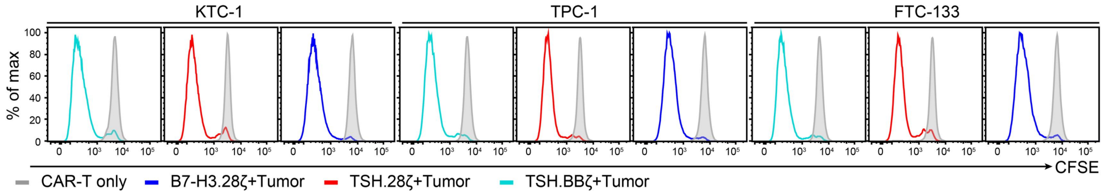

Figure S4 TSH-CAR-T shows a strong expansion ability after being stimulated by tumor cell antigens. The proliferation of CAR-T cells was analyzed by CFSE dilution assay after coculture with THSR-Tg thyroid cancer cells at a ratio of 1:1 for 5 days.

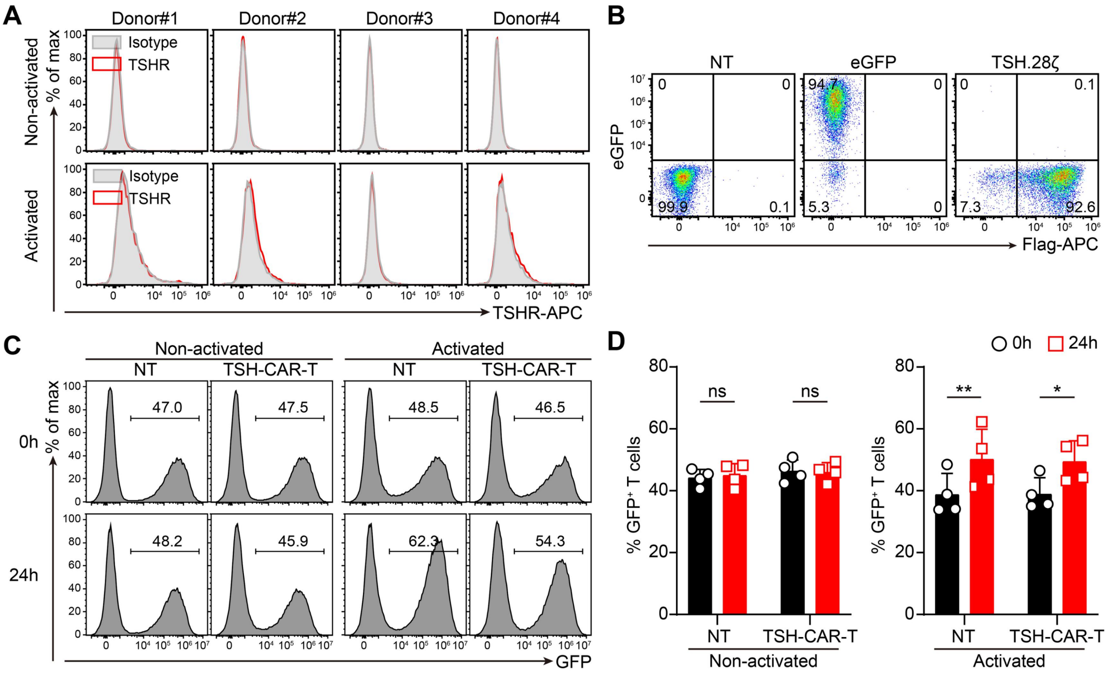

Figure S5. T cells do not express TSHR and TSH-CAR-T cells have no self-killing effect.

(A) T cells from different donors were activated for 24 hours or not activated by CD3 and CD28 antibodies, and the expression of TSHR on T cells was examined by flow cytometry.

(B) Representative flow cytometry plots showing the expression of GFP or CAR (Flag$^+$) in T cells.

(C-D) GFP-labeled activated or non-activated T cells were cocultured with NT or TSH-CAR-T cells from the same donor at a 1:1 ratio. The proportion of GFP$^+$ cells was examined by flow cytometry at the beginning of the coculture and 24 h post-coculture (C); the quantification of residual GFP$^+$ T cells is illustrated (D), n=4. $^*P$ < 0.05 and $^{**}P$ < 0.01; paired *t*-test with two-tailed *p* value calculation.

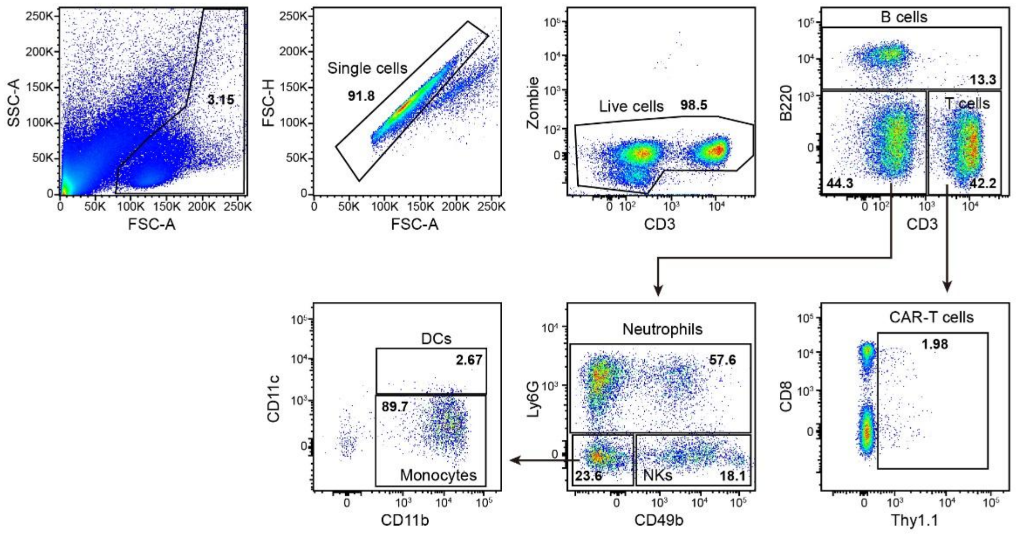

Figure S6. Representative flow plot gating strategy of the cell subsets in the peripheral blood. The different cell subsets in the peripheral blood were analyzed by flow cytometry on day 7, 15, 22 and 30 after CAR-T cells infusion in the safety evaluation syngeneic mouse model. T cells ($CD3^+$), B cells ($B220^+$), CAR-T cells ($CD3^+Thy1.1^+$), Neutrophils ($CD3^-B220^-Ly6G^+$), NKs ($CD49b^+$), DCs ($CD11b^+CD11c^+Ly6G^-$), Monocytes ($CD11b^+Ly6G^-CD11c^-$).

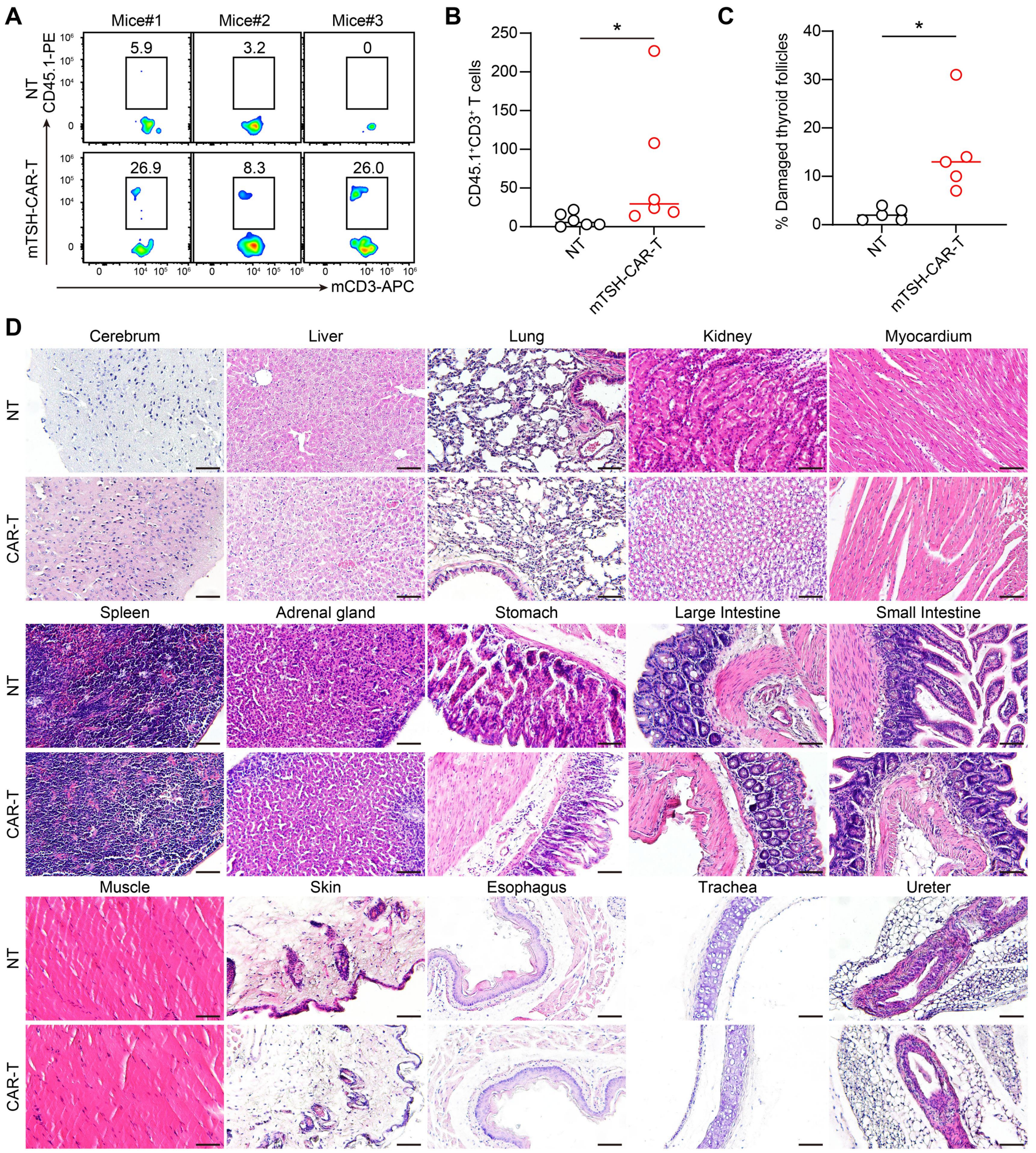

Figure S7. mTSH-CAR-T cells don't exhibit on target/off tumor effect except impairment to thyroid follicles.

(A-B) Representative flow cytometry plots showing the infiltration of CD45.1$^+$ T cells in thyroid tissue (A), and the quantification of CD45.1$^+$ T cell number is illustrated in (B), (n=6). *$P < 0.05$; unpaired and non-parametric Mann-Whitney test with two-tailed p value calculation.

(C) The tissue pathology of thyroid was examined on day 7 post mTSH-CAR-T infusion, and the proportion of damaged thyroid follicles was quantified (n=5/group). *$P < 0.05$; unpaired and non-parametric Mann-Whitney test with two-tailed p value calculation.

(D) Representative HE staining images of the indicated organ sections on day 7 post mTSH-CAR-T infusion. Scale bars are 100 μm.

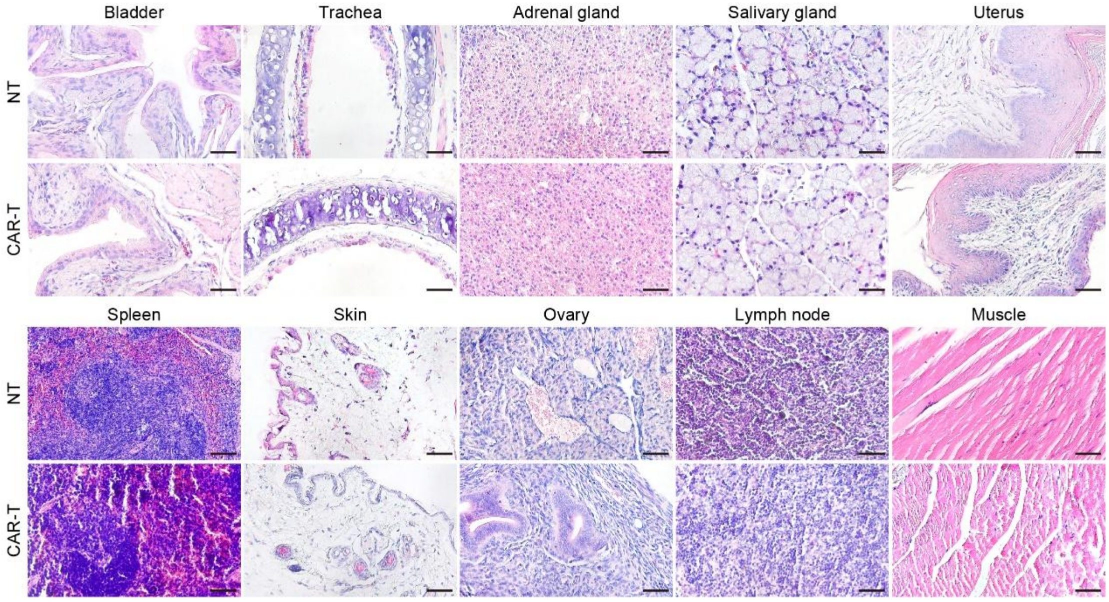

Figure S8. Tissue pathology of major organs in the mice of the safety evaluation experiment. Representative HE staining images of the indicated organ sections at the end of the long-term safety evaluation experiment (day 30) (NT: n=4, mTSH-CAR-T: n=4). Scale bars are 100 μm.

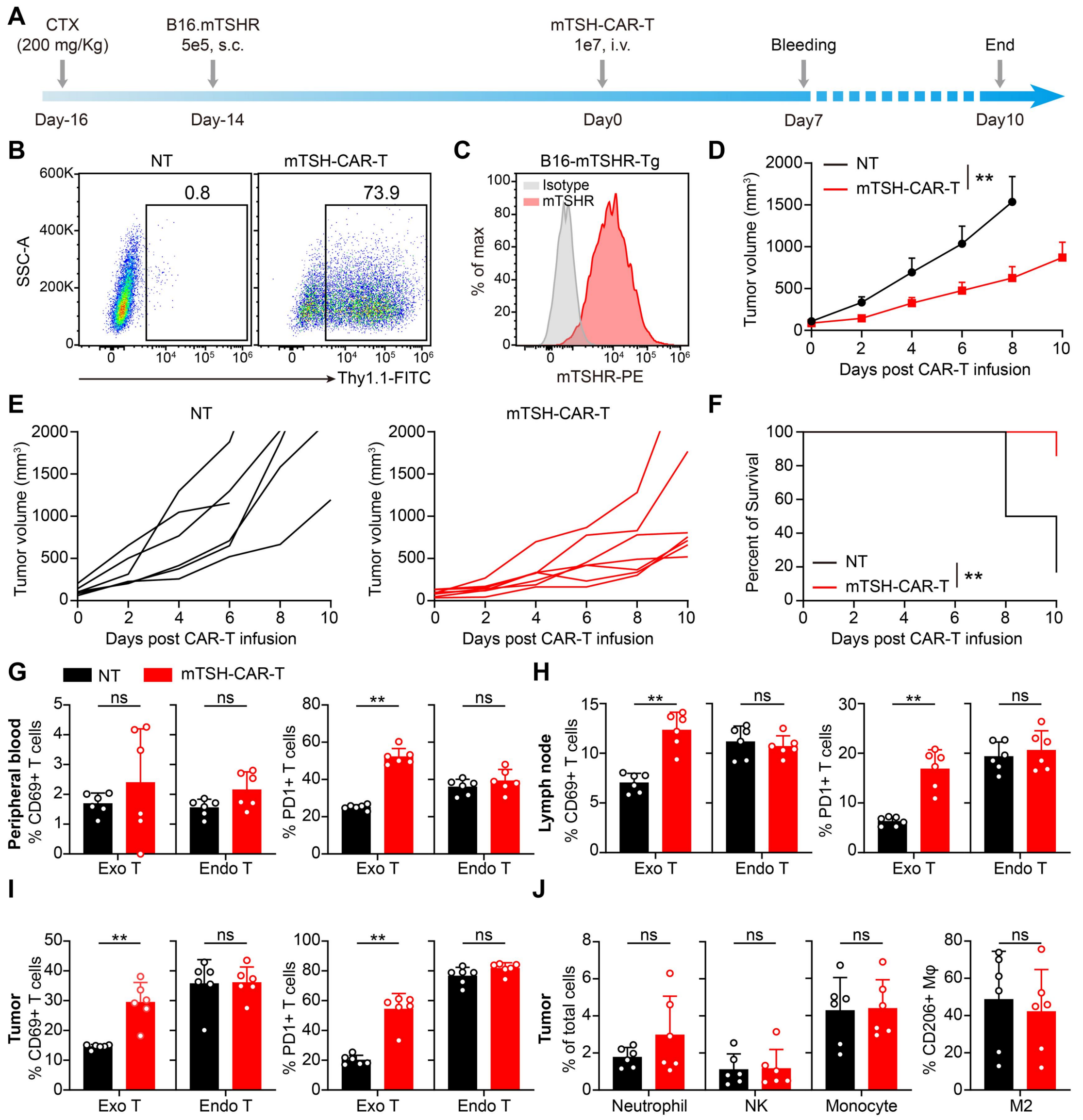

Figure S9. mTSH-CAR-T cells effectively control tumor growth in immunocompetent mice without significantly changing the tumor immune microenvironment.

(A) Schema of the B16-mTSHR syngeneic mouse model. The C57BL/6J mice was pretreated with CTX (200 mg/kg) two days prior the subcutaneous injection of B16-mTSHR cells (5×10^5), 14 days later, treated with 1×10^7 mTSH-CAR-T cells intravenously.

(B) Representative flow cytometry plots showing the transduction efficiency of mTSH-CAR.

(C) Representative flow cytometry plots showing the mTSHR expression pattern in the B16-mTSHR-tg cell line.

(D-E) The tumor growth was monitored every 2 days and presented as mean tumor volume (D) and individual tumor volume of each mouse (E). (NT: n=6; mTSH-CAR-T: n=7). ***P < 0.001; two-way ANOVA with Tukey's multiple comparisons test correction.

(F) Kaplan-Meier survival curve of mice in (D), mice were euthanized when the tumor volume reached 2000 mm^3.

(G-J) In a parallel experiment, each mouse was inoculated with 1×10^6 tumor cells, and treated

with CAR-T cells 7 days post tumor inoculation. Five days after CAR-T infusion (1×10^7), the activation of exogenous (Exo T, $CD3^+CD45.1^+$) and endogenous (Endo T, $CD3^+CD45.1^-$) T cells in the peripheral blood (G), nearby lymph nodes (H) and tumor (I) was examined by measuring the expression of CD69 and PD1 via flow cytometry. The proportion of other immune cells within tumor was also analyzed (J) (NT: n=6, mTSH-CAR-T: n=6). **$P < 0.05$; ns, not significant; unpaired and non-parametric Mann-Whitney test with two-tailed p value calculation.
